# Genome-Wide Identification of Glyoxalase Genes in *Medicago truncatula* and Their Expression Profiling in Response to Various Developmental and Environmental Stimuli

**DOI:** 10.3389/fpls.2017.00836

**Published:** 2017-06-01

**Authors:** Ajit Ghosh

**Affiliations:** Department of Biochemistry and Molecular Biology, Shahjalal University of Science and TechnologySylhet, Bangladesh

**Keywords:** glyoxalase, *Medicago truncatula*, transcript alteration, gene duplication, expression profiling, abiotic stress, salinity, drought

## Abstract

Glyoxalase is an evolutionary highly conserved pathway present in all organisms. Conventional glyoxalase pathway has two enzymes, glyoxalase I (GLYI) and glyoxalase II (GLYII) that act sequentially to detoxify a highly cytotoxic compound methylglyoxal (MG) to D-lactate with the help of reduced glutathione. Recently, proteins with DJ-1/PfpI domain have been reported to perform the same conversion in a single step without the help of any cofactor and thus termed as “unique glyoxalase III” enzyme. Genome-wide analysis of glyoxalase genes have been previously conducted in *Arabidopsis*, rice and Soybean plants, but no such study was performed for one of the agricultural important model legume species, *Medicago truncatula*. A comprehensive genome-wide analysis of *Medicago* identified a total of putative 29 GLYI, 14 GLYII genes, and 5 glyoxalase III (DJ-1) genes. All these identified genes and their corresponding proteins were analyzed in detail including their chromosomal distribution, gene duplication, phylogenetic relationship, and the presence of conserved domain(s). Expression of all these genes was analyzed in different tissues as well as under two devastating abiotic stresses- salinity and drought using publicly available transcript data. This study revealed that *MtGLYI*-4, *MtGLYII*-6, and *MtDJ-1*A are the constitutive members with a high level of expression at all 17 analyzed tissues; while *MtGLYI*-1, *MtGLYI*-11, *MtGLYI*-5, *MtGLYI-*7, and *MtGLYII-*13 showed tissue-specific expression. Moreover, most of the genes displayed similar pattern of expression in response to both salinity and drought stress, irrespective of stress duration and tissue type. *MtGLYI*-8, *MtGLYI*-11, *MtGLYI*-6, *MtGLYI*-16, *MtGLYI*-21, and *MtGLYII-*9 showed up-regulation, while *MtGLYI*-17 and *MtGLYI*-7/9 showed down-regulation in response to both stresses. Interestingly, *MtGLYI*-14/15 showed completely opposite pattern of expression in these two stresses. This study provides an initial basis about the physiological significance of glyoxalase genes in plant development and stress response of *Medicago* that could be explored further.

## Introduction

**FIGURE 1:**
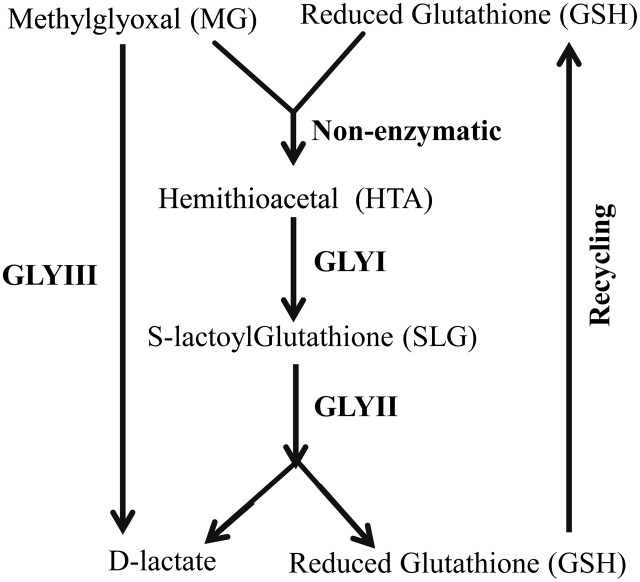
**Schematic diagram of glyoxalase pathway.** Conversion of MG into D-lactate could be performed in two different ways. First one, conventional glyoxalase pathway consists of two enzymes, GLYI and GLYII with an intermediate product of *S*-D-lactoylglutathione (SLG). The second and unique one is glyoxalase III (GLYIII) in a single step without the help of any cofactor.

Glyoxalase pathway is one of the evolutionary highly conserved pathways which have been found in all era of organisms starting from lower prokaryotic *Escherichia coli* to higher eukaryotes *Homo sapiens* (Kaur et al., 2014a). Glyoxalase pathway detoxifies highly cytotoxic compound MG into its non-toxic form, D-lactate (**Figure 1**). Conventional glyoxalase pathway consists of two enzymes; GLYI and GLYII (Kaur et al., 2014c). MG and GSH generate a non-enzymatic adduct hemithioacetal (HTA). GLYI isomerizes HTA to *S*-lactyl-glutathione (SLG) which is further hydrolysed by GLYII into D-lactate and GSH (**Figure 1**). Recently, there are some reports of the presence of another unique glyoxalase member named Glyoxalase III (GLYIII) (Lee et al., 2012; Kwon et al., 2013; Ghosh et al., 2016). GLYIII could do the same conversion of MG into D-lactate in a single step (**Figure 1**).

Besides its proposed metabolic role of MG metabolism, glyoxalase enzymes have been found to be involved in various other cellular functions. Various complications of diabetes, such as nephropathy, retinopathy, neuropathy, and cardiovascular complications have been protected by glyoxalase system (Rabbani and Thornalley, 2011). Glyoxalase enzymes have also been reported to be involved in cell division and proliferation, and microtubule assembly of human (Thornalley, 1990). Thus, this pathway has been considered as “marker for cell growth and division.” Similarly, numerous studies suggested its potential role in stress tolerance pathways of plant (Kaur et al., 2014a). Transgenic plants over-expressing either *GLYI* or *GLYII* were found to provide significant tolerance against multiple abiotic stresses including salinity, drought, and heavy metal toxicity in various plant species (Singla-Pareek et al., 2003; Ghosh et al., 2014; Kaur et al., 2014a; Mustafiz et al., 2014). Moreover, overexpression of *GLYI* and *GLYII* genes together in transgenic tobacco and tomato plants confers extensive salt and heavy metal tolerance by maintaining the level of MG normal and decreasing oxidative stresses (Singla-Pareek et al., 2003, 2006; Alvarez Viveros et al., 2013). Thus MG and glyoxalases are considered as the potential biomarker for plant stress physiology (Kaur et al., 2014c).

Glyoxalase proteins have been extensively analyzed from various microorganisms and animal systems such as *E. coli*, *H. sapiens*, *Saccharomyces cerevisiae* (Kaur et al., 2013). In spite of great importance, very little study has been conducted on plant glyoxalases as compared to non-plant species. Plant glyoxalase activity has been first reported from Douglas fir needles by Smits and Johnson (1981). Presently, the presence of glyoxalase genes and corresponding enzyme activity has been studied from various plant species, such as rice, *Arabidopsis*, tomato, tobacco, soybean, wheat, *Zea mays*, sugarcane, *Brassica* etc. (Kaur et al., 2014a,c). The activity of plant GLYI has been reported to be dependent on either Ni^2+^ or Zn^2+^ (Jain et al., 2016). Plant GLYII enzymes possess various metals such as Fe^2+^, Zn^2+^, Mn^2+^ in their structure and these metal ions are essential for its optimum activity (Ghosh et al., 2014). However, the activity of GLYIII enzyme from *E. coli* has been reported to be metal independent (Subedi et al., 2011).

Most of the plant genes exist as a family due to the genome expansion and gene duplication events occurred during the evolution of plant (Guo, 2013). Availability of complete genome sequence databases of various plants species provides the opportunity to identify plants gene families. *In silico* genome-wide analyses of *Arabidopsis thaliana*, *Glycine max*, and *Oryza sativa* has been carried out to identify glyoxalase gene families (Mustafiz et al., 2011; Ghosh and Islam, 2016; Ghosh et al., 2016). There are 11 *GLYI* and 5 *GLYII* genes in *Arabidopsis*; 24 *GLYI* and 12 *GLYII* genes in *G. max*; and 11 potential *GLYI* and three *GLYII* genes in rice (Mustafiz et al., 2011; Ghosh and Islam, 2016). Expression profiling of all these genes has been performed at different developmental stages, tissues, and abiotic stresses using publicly available microarray database. It has been observed that *AtGLYI-3*, *AtGLYII-2*, and *AtGLYII-5* expressed constitutively in all the tissues and developmental stages; while *AtGLYI-7* and *AtGLYII-2* are the most stress-inducible members (Mustafiz et al., 2011). In *G. max*, *GmGLYI-7* and *GmGLYII-8* showed maximum expression in all the developmental stages and tissues; while *GmGLYI-6*, *GmGLYI-9*, *GmGLYI-20*, *GmGLYII-5*, and *GmGLYII-10* were highly abiotic stress responsive members (Ghosh and Islam, 2016). Similarly, *OsGLYI-11, OsGLYII-2*, and *OsGLYII-3* expressed constitutively; while *OsGLYI-11* and *OsGLYII-3* were the most stress responsive members (Mustafiz et al., 2011).

In spite of having a handful genome sequence data deposited in the publicly available database, genome-wide analysis of glyoxalase gene families was not performed in *Medicago truncatula. M. truncatula* is considered as an excellent model plant due to its relatively small genome size and very high genetic transformation efficiency (Zhang et al., 2013). Moreover, this is a symbiotic legume plant that fixed 40–60 million tons of N_2_ per annum (Smil, 1999), is equivalent to US$7 to 10 billion (Graham and Vance, 2003). A detailed genome-wide identification of *Medicago* conventional (*GLYI* and *GLYII*) and unique glyoxalase (DJ-1) genes is presented along with their phylogenetic relationship, chromosomal distribution, gene and protein structure, genome duplication and transcript profiling. Results indicate the presence of 29 GLYI, 14 GLYII, and 5 DJ-1 genes in *Medicago* that codes for 35, 27, and 6 proteins, respectively. This is the largest glyoxalase protein family reported to date in any organism. Expression analysis of these genes based on publicly available microarray data indicates the differential regulation of glyoxalase family members depending on developmental and environmental cues. In particular, *MtGLYI*-8, *MtGLYII*-9, and *MtDJ-*1C are found to be the most up-regulated stress responsive members that might play an imperative role to minimize the accumulation of MG under stress. This study provides a strong base for further characterization and functional validation of glyoxalase genes from *M. truncatula*.

## Materials and Methods

### Identification and Nomenclature of *Glyoxalase* Genes in *Medicago truncatula*

Putative *Medicago* GLYI, GLYII and unique GLYIII (DJ-1) proteins were identified by BLASTP search at JCVI *M. truncatula* annotation database 4.0v1^1^ (Young et al., 2011) with an *e*-value of 1 using previously reported *Arabidopsis* GLYI (GenBank: AEE28797.1), *Brassica* GLYII (GenBank: AAO26580.1) and rice DJ-1C (LOC_Os04g57590) protein sequence as query, respectively. Each of the identified sequences was used as secondary query subsequently until finding new members. Protein sequences of all BLAST hits were retrieved from the database and analyzed using the Hidden Markov Model (HMM) profile of Pfam^2^ with *e*-value of 1. The presence of glyoxalase domain (PF00903) or Metallo-beta-lactamase domain (PF00753) or DJ-1/PfpI domain (PF01965) confirmed the identity of the proteins as GLYI or GLYII or DJ-1, respectively. Identified proteins were nomenclature as prefix “Mt” for *M. truncatula*, followed by GLYI or GLYII or DJ-1. They have numbered arabically for GLYI and GLYII, or alphabetically for DJ-1 depending on their chromosomal position as mentioned in previous studies (Ghosh and Islam, 2016; Ghosh et al., 2016). Detailed information about the identified genes and corresponding proteins was retrieved using locus search option of *M. truncatula* annotation database 4.0v1^1^ (Young et al., 2011). Sub-cellular localization of all these identified proteins was predicted using CELLO v.2.5: sub-cellular localization predictor^3^ (Yu et al., 2006), Wolf PSORT^4^ (Horton et al., 2007) and ChloroP^5^ (Emanuelsson et al., 1999) tools with default parameters (**Tables 1**–**3**).

**Table 1:** List of putative Glyoxalase I gene family members of *Medicago truncatula.*

Gene name	Locus name	Transcripts	Coordinate (5′–3′)	Transcript length (bp)	CDS (bp)	Protein			Localization
							
						Length (aa)	MW (kDa)	pI	
*MtGLYI-1*	Medtr1g022325	Medtr1g022325.1	7056631–7055156	1683	1287	428	47.38	5.66	Ch^a,b^, Cy^a,b^
*MtGLYI-2*	Medtr1g115170	Medtr1g115170.1	51845699–51846145	–	447	148	16.76	4.69	Cy^a,b^
*MtGLYI-3*	Medtr2g005880	Medtr2g005880.1	372509–376355	4086	546	181	20.60	5.26	ExC^a^, Cy^a^, Ch^b^
*MtGLYI-4*	Medtr2g023500	Medtr2g023500.1	8303233–8298020	5211	864	287	32.10	5.26	Cy^a,b^
*MtGLYI-5*	Medtr2g103460	Medtr2g103460.1	44546518–44544702	2214	516	171	19.16	5.93	Cy^a,b^
*MtGLYI-6*	Medtr2g103490	Medtr2g103490.1	44556161–44555344	815	516	171	19.42	5.04	Cy^a,b^, Nu^b^
*MtGLYI-7*	Medtr3g110185	Medtr3g110185.1	51187554–51183426	4129	2466	821	92.26	8.06	Nu^a^, Cy^b^
*MtGLYI-8*	Medtr4g011020	Medtr4g011020.1	2571851–2573439	1992	528	175	20.01	5.87	Cy^a,b^, Nu^b^
*MtGLYI-9*	Medtr4g039890	Medtr4g039890.1	14172120–14173530	1411	744	247	27.56	7.33	Cy^a,b^
*MtGLYI-10*	Medtr4g057685	Medtr4g057685.1	21216977–21211083	8202	705	234	26.69	9.49	Mt^a^, Ch^b,c^
		Medtr4g057685.2	21216757–21211083	8202	561	186	20.95	5.33	Cy^a^, Nu^b^
*MtGLYI-11*	Medtr4g063500	Medtr4g063500.1	23518703–23522474	4097	426	141	15.88	6.24	Cy^a,b^
		Medtr4g063500.2	23518703–23522474	4097	387	128	14.40	6.52	Cy^a,b^
*MtGLYI-12*	Medtr4g114080	Medtr4g114080.1	46953345–46952651	1028	507	168	19.23	5.13	ExC^a^, Nu^a^, Ch^b^
*MtGLYI-13*	Medtr4g125860	Medtr4g125860.1	52258825–52261268	3392	582	193	21.65	7.32	Mt^a,b^, Ch^b,c^
*MtGLYI-14*	Medtr4g132260	Medtr4g132260.1	55265824–55262670	3155	846	281	31.47	4.73	Cy^a,b^
*MtGLYI-15*	Medtr4g132270	Medtr4g132270.1	55272349–55269373	2977	915	304	33.95	4.98	Cy^a,b^
		Medtr4g132270.2	55271711–55269373	2977	846	281	31.55	4.88	Cy^a,b^
*MtGLYI-16*	Medtr5g006360	Medtr5g006360.1	881390–880412	1234	423	140	15.86	6.35	Cy^a^, Nu^b^
*MtGLYI-17*	Medtr5g006370	Medtr5g006370.1	883839–883046	1856	420	139	15.66	5.48	Nu^a^, Mt^a^, Cy^a^, Ch^b^
		Medtr5g006370.2	883585–883046	1856	336	111	12.53	5.11	Mt^a^, Cy^a,b^
		Medtr5g006370.3	883839–883162	1856	390	129	14.59	6.3	Nu^a^, Mt^a^, Cy^b^
*MtGLYI-18*	Medtr5g009740	Medtr5g009740.1	2433440–2432537	1209	351	116	12.82	7.57	Mt^a^, Ch^b^
*MtGLYI-19*	Medtr5g021610	Medtr5g021610.1	8378105–8376835	1446	624	207	23.91	4.7	ExC^a^, Nu^a,b^
*MtGLYI-20*	Medtr5g090990	Medtr5g090990.1	39642769–39640064	3139	1287	428	47.37	5.61	Ch^a,b^, Cy^a^, Nu^b^
*MtGLYI-21*	Medtr5g091060	Medtr5g091060.1	39679453–39682352	3349	1314	437	48.36	5.34	Ch^a,b,c^
*MtGLYI-22*	Medtr6g087120	Medtr6g087120.1	33502501–33497925	5130	1047	348	39.15	7.96	Mt^a^, Ch^b,c^
*MtGLYI-23*	Medtr8g076160	Medtr8g076160.1	32249509–32248727	1060	582	193	21.94	5.04	ExC^a,b^, Cy^a^
*MtGLYI-24*	Medtr8g102980	Medtr8g102980.1	43369583–43365194	4842	1044	347	38.82	7.03	Ch^a,b,c^
		Medtr8g102980.2	43369583–43366886	4842	1029	342	38.18	7.86	Ch^a,b,c^
*MtGLYI-25*	Medtr0003s0630	Medtr0003s0630.1	351938–354470	2533	603	200	23.18	4.86	Nu^a^, Cy^a,b^
*MtGLYI-26*	Medtr0430s0010	Medtr0430s0010.1	826–1190	365	258	85	9.63	9.30	Cy^a^
*MtGLYI-27*	Medtr1239s0010	Medtr1239s0010.1	34–1801	1768	1681	560	62.66	6.24	Cy^a,b^
*MtGLYI-28*	Medtr1275s0010	Medtr1275s0010.1	9–1946	1938	1548	515	55.66	8.22	Mt^a^, Cy^b^
*MtGLYI-29*	Medtr1759s0010	Medtr1759s0010.1	211–1341	1128	987	328	35.34	9.62	Ch^a^, ExC^b^


*bp base pair; MW, molecular weight; kDa, kilo Dalton; Ch, chloroplast; Cy, cytosol; Mt, mitochondria; ExC, extra cellular; Nu, nuclear. ^a^Localization prediction by CELLO v.2.5 (http://cello.life.nctu.edu.tw/). ^b^Localization prediction by pSORT (http://www.genscript.com/wolf-psort.html). ^c^Chloroplast localization signal confirmed by ChloroP (http://www.cbs.dtu.dk/services/ChloroP/).*

**Table 2:** List of putative Glyoxalase II gene family members of *Medicago truncatula.*

Gene name	Locus name	Transcripts	Coordinate (5′–3′)	Transcript length (bp)	CDS (bp)	Protein			Localization
							
						Length (aa)	MW (kDa)	pI	
*MtGLYII-1*	Medtr1g032500	Medtr1g032500.1	11561502–11550336	11903	1605	534	60.13	6.73	Cy^a,b^
		Medtr1g032500.2	11561502–11551704	11903	1248	415	47.02	7.58	Cy^a,b^
*MtGLYII-2*	Medtr1g050492	Medtr1g050492.1	19478035–19486897	8863	2223	740	82.00	4.82	Cy^a,b^, Ch^b^
*MtGLYII-3*	Medtr1g110300	Medtr1g110300.1	49751952–49742911	9300	1572	523	58.66	6.9	Cy^a^, Ch^b^
		Medtr1g110300.2	49751952–49743319	9300	1461	486	54.32	6.03	Cy^a^, Ch^b^
		Medtr1g110300.3	49751952–49745653	9300	1116	371	41.53	5.84	Cy^a^, Ch^b^
		Medtr1g110300.4	49751952–49745288	9300	1431	476	53.22	6.2	Cy^a^, Ch^b^
		Medtr1g110300.5	49751952–49743319	9300	1557	518	58.30	6.12	Cy^a^, Ch^b^
		Medtr1g110300.6	49751952–49745288	9300	1527	508	57.20	6.28	Cy^a^, Ch^b^
		Medtr1g110300.7	49751952–49745802	9300	1155	384	42.64	5.62	Cy^a^, Ch^b^
*MtGLYII-4*	Medtr2g006180	Medtr2g006180.1	489282–485609	4028	2073	690	76.96	6.37	Cy^a^,^b^
*MtGLYII-5*	Medtr2g018660	Medtr2g018660.1	5892053–5896603	5924	963	320	35.87	6.18	Nu^a^, Cy^b^
*MtGLYII-6*	Medtr2g072190	Medtr2g072190.1	30330754–30334767	4591	858	285	31.43	8.42	Mt^a,b^
		Medtr2g072190.2	30330754–30333959	4591	723	240	26.47	8.93	Mt^a,b^, Ch^b,c^
		Medtr2g072190.3	30330754–30334011	4591	780	259	28.77	7.72	Mt^a,b^, Ch^b,c^
*MtGLYII-7*	Medtr2g099090	Medtr2g099090.1	42458819–42455183	4198	990	329	36.10	8.65	Mt^a^, Ch^b,c^
		Medtr2g099090.2	42458690–42455183	4198	942	313	34.51	8.57	Mt^a^, Ch^b,c^
*MtGLYII-8*	Medtr2g101390	Medtr2g101390.1	43590697–43596569	6147	2853	950	104.98	6.29	Nu^a^, Cy^a^, Mt^a^, Ch^b,c^
*MtGLYII-9*	Medtr3g089020	Medtr3g089020.1	33966445–33957865	9309	2604	867	96.36	8.11	Ch^a,b,c^
*MtGLYII-10*	Medtr4g068100	Medtr4g068100.1	25474066–25470040	4584	948	315	34.99	6.78	Mt^a^, Nu^b^
		Medtr4g068100.2	25472955–25470040	4584	762	253	28.03	5.55	Cy^a^,^b^
		Medtr4g068100.3	25474066–25470489	4584	906	301	33.49	6.78	Mt^a^, Pm^a^, ExC^a^, Nu^b^
		Medtr4g068100.4	25474066–25470040	4584	972	323	35.86	8.14	Mt^a^, Nu^b^
*MtGLYII-11*	Medtr4g103770	Medtr4g103770.1	42917141–42924712	7724	2166	721	80.73	9.83	Mt^a^, Ch^b,c^
*MtGLYII-12*	Medtr5g068440	Medtr5g068440.1	28969883–28974477	5037	777	258	28.53	5.34	Cy^a^,^b^
*MtGLYII-13*	Medtr8g017270	Medtr8g017270.1	5810801–5804905	6295	1026	341	38.00	7.68	Ch^a,b,c^, Mt^a^, Pm^a^
*MtGLYII-14*	Medtr2166s0010	Medtr2166s0010.1	62–925	861	864	287	31.26	6.6	ExC^a^, Cy^a^, Ch^b^


*Bp, base pair; MW, molecular weight; kDa, kilo Dalton; Ch, chloroplast; Cy, cytosol; Mt, mitochondria; ExC, extra cellular; Nu, nuclear. ^a^Localization prediction by CELLO v.2.5 (http://cello.life.nctu.edu.tw/). ^b^Localization prediction by pSORT (http://www.genscript.com/wolf-psort.html). ^c^Chloroplast localization signal confirmed by ChloroP (http://www.cbs.dtu.dk/services/ChloroP/).*

**Table 3:** List of putative Glyoxalase III gene family members of *Medicago truncatula.*

Gene name	Locus name	Transcripts	Coordinate (5′–3′)	Transcript length (bp)	CDS (bp)	Protein			Localization
							
						Length (bp)	MW (kDa)	pI	
*Mt DJ-1A*	Medtr2g078060	Medtr1g022325.1	32401642–32405412	3968	1287	428	46.87	7.25	Ch^a,b,c^
*Mt DJ-1B*	Medtr3g064093	Medtr3g064093.1	28828715–28828368	510	279	92	10.43	6.33	ExC^a^, Pm^a^, Cy^b^
*Mt DJ-1C*	Medtr3g064115	Medtr3g064115.1	28837989–28837425	626	285	94	10.25	6.77	ExC^a^, Pm^a^, Cy^a,b^
*Mt DJ-1D*	Medtr3g064140	Medtr3g064140.1	28843504–28840287	3588	1164	387	41.41	5.97	Cy^a,b^
*MtDJ-1E*	Medtr4g085900	Medtr4g085900.1	33593600–33596516	3437	1356	451	48.20	9.42	Mt^a^, Ch^a,b,c^
		Medtr4g085900.2	33593600–33595499	3437	1014	337	36.25	6.95	Pm^a^, Mt^a,b^, Ch^a,b,c^


*Bp, base pair; MW, molecular weight; kDa, kilo Dalton; Ch, chloroplast; Cy, cytosol; Mt, mitochondria; ExC, extra cellular; Nu, nuclear; Pm, plasma membrane. ^a^Localization prediction by CELLO v.2.5 (http://cello.life.nctu.edu.tw/). ^b^Localization prediction by pSORT (http://www.genscript.com/wolf-psort.html). ^c^Chloroplast localization signal confirmed by ChloroP (http://www.cbs.dtu.dk/services/ChloroP/).*

### Chromosomal Localization and Gene Duplications

**FIGURE 2:**
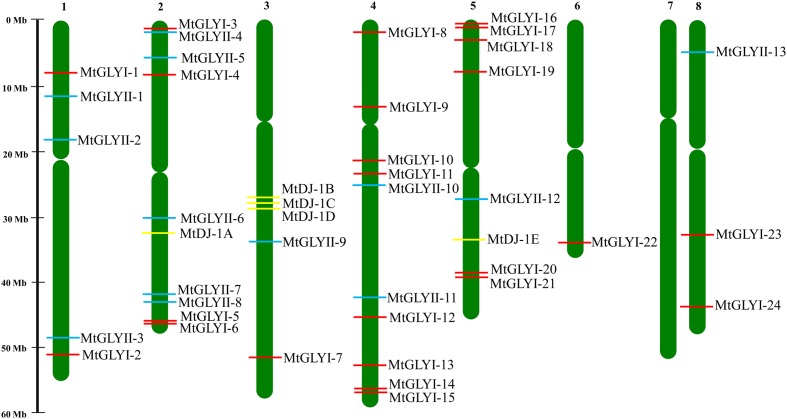
**Chromosomal distribution glyoxalase genes in *Medicago truncatula*.** Presence and position of all identified conventional and unique glyoxalase genes were shown in seven different chromosomes. Chromosome numbers are indicated at the top of each bar. Chromosome 7 did not have any glyoxalase genes. The position of GLYI, GLYII, and DJ-1 genes are marked with red, cyan, and yellow color boxes. The name of each gene is mentioned just right side of the colored boxes.

Chromosomal location for all the newly identified *glyoxalase* genes was pointed using genome browser of *M. truncatula* database 4.0v1^1^ (Young et al., 2011) (**Figure 2**). Duplication events between the *Medicago* glyoxalase genes with that of *Medicago*, *Arabidopsis*, rice, and soybean were identified using plant genome duplication database^6^ analysis (Lee et al., 2013). The rate of synonymous (*K*_s_) and non-synonymous substitution (*K*_a_) was retrieved from plant genome duplication database, too (**Table 4**).

**Table 4:** List of glyoxalase gene duplication events between *Medicago truncatula, Arabidopsis thaliana, Oryza sativa*, and *Glycine max.*

	*Orthologous genes*												*Paralogous genes*			
	
	*Arabidopsis thaliana*				*Oryza sativa*				*Glycine max*				*Medicago truncatula*			
				
Locus 1	Locus 2	*K*_a_	*K*_s_	*K*_a_/*K*_s_	Locus 2	*K*_a_	*K*_s_	*K*_a_/*K*_s_	Locus 2	*K*_a_	*K*_s_	*K*_a_/*K*_s_	Locus 2	*K*_a_	*K*_s_	*K*_a_/*K*_s_
*MtGLYI-4*	*AtGLYI-3*	0.13	1.61	0.08									*MtGLYI-14*	0.14	0.51	0.27
*MtGLYI-5*	*AtGLYI-4*	0.25	0	-	*OsGLYI-1*	0.38	0	-					*MtGLYI-8*	0.19	1.29	0.15
*MtGLYI-7*													*MtGLYI-22*	0.29	2.15	0.13
*MtGLYI-10*	*AtGLYI-2*	0.24	0	-					*GmGLYI-16*	0.09	0.67	0.13				
*MtGLYI-11*					*OsGLYI-3*	0.21	0	-								
*MtGLYI-12*	*AtGLYI-4*	0.31	0	-	*OsGLYI-1*	0.37	0	-					*MtGLYI-8*	0.25	0	-
*MtGLYI-13*	*AtGLYI-11*	0.24	3.39	0.07					*GmGLYI-23*	0.09	0.36	0.25				
*MtGLYI-18*	*AtGLYI-10*	0.14	1.64	0.09												
*MtGLYI-19*													*MtGLYI-23*	0.12	0.92	0.13

*MtGLYII-3*									*GmGLYII-12*	0.13	0.44	0.29				
*MtGLYII-6*	*AtGLYII-2*	0.18	0	-					*GmGLYII-6*	0.11	0.56	0.19				
*MtGLYII-7*	*AtGLYII-1*	0.21	1.86	0.11	*OsGLYII-3*	0.28	0	-	*GmGLYII-7*	0.08	0.39	0.21	*MtGLYII-10*	0.18	0.7	0.26
*MtGLYII-10*	*AtGLYII-4*	0.27	1.89	0.14	*OsGLYII-3*	0.34	5.04	0.07	*GmGLYII-9*	0.16	0.65	0.25				

*MtDJ-1A*	*AtDJ-1A*	0.29	1.64	0.17					*GmDJ-1E*	0.11	0.53	0.21				
*MtDJ-1B*									*GmDJ-1C*	0.48	0.80	0.6				
*MtDJ-1E*	*AtDJ-1C*	0.34	1.39	0.24					*GmDJ-1B*	0.13	0.65	0.19	


### Multiple Sequence Alignment and Phylogenetic Analysis

**FIGURE 3:**
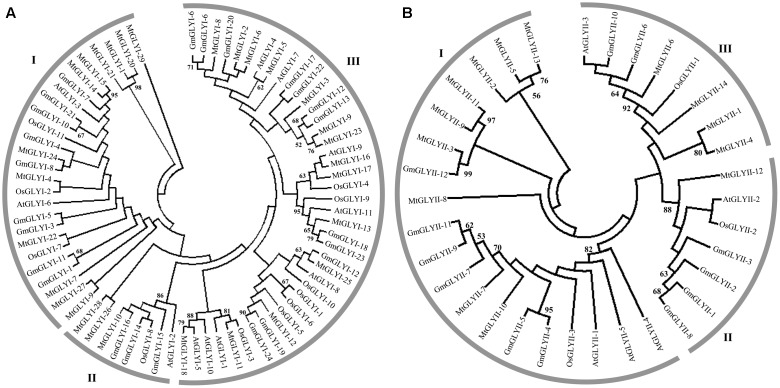
**Phylogenetic relationships of *Medicago*, *Arabidopsis*, rice, and soybean Glyoxalase proteins.** Phylogenetic analysis of GLYI **(A)** and GLYII **(B)** proteins were performed separately using all the reported members from *Medicago*, *Arabidopsis*, rice, and soybean. The tree was constructed using full-length amino acid sequences based on a neighbor-joining method with 1000 bootstrap value.

Phylogenetic relationship among glyoxalase proteins from *Arabidopsis*, rice, soybean, and *Medicago* were investigated using MEGA 7.0 (Kumar et al., 2016) with the Neighbor-Joining method and 1000 bootstrap replicates (**Figures 3**, **8A**). Multiple sequence alignment was performed using ClustalW (Larkin et al., 2007) (**Figures 5**, **7**, **8C**). Protein sequences used in the phylogenetic analysis are provided as **Additional Files S1, S2, S3** GLYI, GLYII and DJ-1 proteins, respectively.

### Domain Architecture of MtGLYI, MtGLYII, and MtDJ-1 Proteins

**FIGURE 4:**
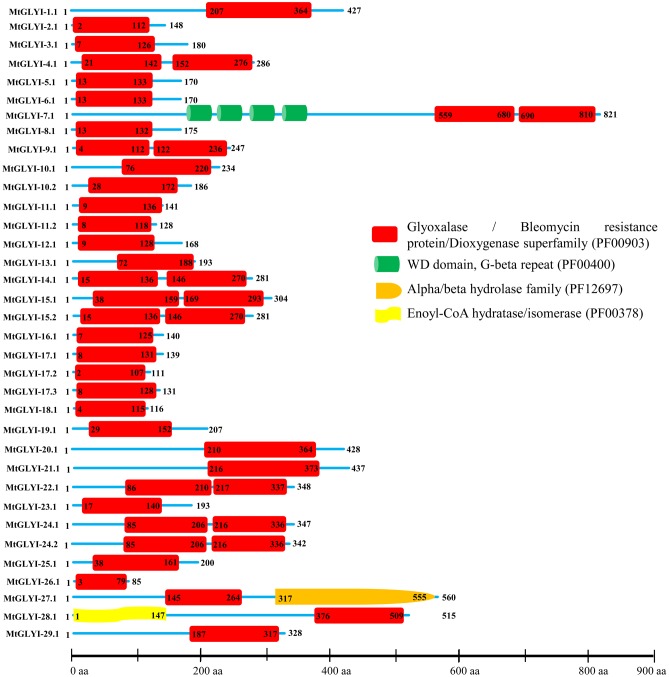
**Schematic representation of MtGLYI proteins structure.** Protein structures of 35 MtGLYI proteins are shown along with the names of all members on the left side of the figure. All MtGLYI proteins were analyzed using Pfam (http://www.sanger.ac.uk/Software/Pfam) for the presence of functional domain(s). All these proteins possess conserved glyoxalase domain (PF00903) along with some other domains such as WD domain, G-beta repeat (PF00400), Alpha/beta hydrolase family (PF12697) and Enoyl-CoA hydratase/isomerase (PF00378). Different domains are indicated by different colored artworks denoted at the right corner. Exact position and number of each domain are indicated by the amino acid number inside the box. The length of protein is also indicated by exact amino acid numbers and relative position of the domains could be interpreted by the scale given below.

All the predicted MtGLYI (35), MtGLYII (27), and MtDJ-1 (6) proteins were analyzed using Pfam^2^ (Finn et al., 2016) to confirm the presence of their respective conserved domains and their exact positions. The position of glyoxalase domain (PF00903) or Metallo-beta-lactamase domain (PF00753) or DJ-1/PfpI domain (PF01965) were identified for all GLYI or GLYII or DJ-1 proteins and the relative figure was generated (**Figures 4**, **6**, **8B**). The presence of some additional domains along with the conserved domain was marked in the corresponding figure at an approximate position.

### Expression Profiling of Glyoxalase Genes at Different Tissues of *M. truncatula*

Genome-wide microarray data of conventional and unique glyoxalase genes were obtained from the *M. truncatula* gene expression Atlas (MtGEA) Project database^7^ (Benedito et al., 2008). The corresponding probesets for all *MtGLYI, MtGLYII*, and *MtDJ-1* genes were identified using NetAffx Analysis Center online Probe Match tool^8^ with default parameters based on respective CDS sequence as query. Genes with more than one probe, probe with the highest expression value was considered for analysis; and single probe for many genes was considered as the same transcriptional profile. Normalized transcript data was obtained for 17 different tissues, including underground tissues-root and nodule; aerial tissues- stem, vegetative bud, petiole, leaf, flower, pod; seed development (seed of 10, 12, 16, 20, 24, and 36 days); and nod development (nod of 4, 10, and 14 days). These normalized expression data was used to generate heatmap (**Figure 9**) and hierarchically clustering based on the Manhattan correlation with average linkage using the Institute for Genomic Research MeV software package^9^ (Saeed et al., 2006).

### Microarray-Based Expression Analysis of *Medicago glyoxalase* Genes in Response to Salinity and Drought

Normalized and curated perturbation expression data of *Medicago glyoxalase* genes were retrieved from Genevestigator Affymetrix *Medicago* genome array^10^ (Hruz et al., 2008; Zimmermann et al., 2008) using the already identified corresponding probe. The log_2_ fold change ratio was retrieved using Genevestigator with default parameters using experiments id: MT-00011: Effects of salt stress on *M. truncatula* seedlings (GSE14029)^11^ and MT-00013: Global reprogramming of transcription and metabolism in *M. truncatula* during progressive drought and after rewatering (E-MTAB-2681) (Zhang et al., 2014). Detailed informations about the gene expression experimental design is provided as **Additional File S4**. These experiments were performed with minimum three replicates and fold change values were calculated in comparison to the untreated control 0 h seedlings in case of salinity and an untreated 2 days control sample of respective tissue in the case of drought. Generation of heat maps (**Figure 10**) and hierarchical clustering with Manhattan distance metric method was performed using Institute for Genomic Research MeV software package^9^ (Saeed et al., 2006).

## Results

### Identification of Glyoxalase Proteins in *Medicago truncatula*

Glyoxalase proteins have been well characterized from various plant as well as animal species. The genome-wide analysis identified a total of 35 unique *Medicago* GLYI proteins encoded by 29 *GLYI* genes. These genes were found to be located on different chromosomes of *Medicago* (**Figure 2**). They were nomenclature as *MtGLYI*-1 to *MtGLYI*-29 according to the order of their chromosomal location (**Table 1**). The number of *Medicago GLYI* genes was found to be greater than previously reported *Arabidopsis* (10), rice (11), and soybean (24) *GLYI* genes (Mustafiz et al., 2011; Ghosh and Islam, 2016). Similarly, a total of 27 *Medicago* GLYII proteins encoded by 14 *GLYII* genes were identified. Both the number of GLYII genes and proteins were found to be greater than previously reported *Arabidopsis* (5 genes and 9 proteins), rice (3 genes and 4 proteins), and soybean (12 genes and 23 proteins) (Mustafiz et al., 2011; Ghosh and Islam, 2016). They were symbolized as *MtGLYII*-1 to *MtGLYII*-14 like *MtGLYI* genes (**Table 2**).

In the same way, genome-wide searching of unique GLYIII proteins in *Medicago* identified six DJ-1 proteins encoded by five genes. They were named as *MtDJ*-1A to *MtDJ-*1E like previously reported members from other species (Ghosh et al., 2016) (**Table 3**). Although the number of genes was almost similar, but the number of MtDJ-1 proteins was less than previously reported *Arabidopsis* (6 genes and 11 proteins), rice (6 genes and 12 proteins) (Ghosh et al., 2016) and soybean (7 genes and 11 proteins) (unpublished data) counterparts. In all cases of *MtGLYI, MtGLYII*, and *MtDJ-1* families, proteins number was found to be greater than that of genes (**Tables 1**–**3**), that indicate the presence of alternate splicing event in *Medicago* glyoxalase transcripts.

### Detailed Analysis of Newly Identified MtGLYI, MtGLYII, and MtDJ-1 Members

All the newly identified glyoxalase members were analyzed further in terms of the length of transcripts, coding DNA sequence (CDS), and polypeptide; molecular weight, isoelectric point and sub-cellular localization of the corresponding protein. CDS length of Mt*GLYI* members was found to be fluctuating from 258 bp (*MtGLYI-26*) to 2466 bp (*MtGLYI-7*) with an average of 1533 bp. Thus, the largest member of MtGLYI protein family is MtGLYI-7 with a polypeptide length of 821 aa and molecular weight of 92.26 kDa. Subsequently, the smallest member is MtGLYI-26 with 85 aa in length and 9.63 kDa in molecular weight (**Table 1**). MtGLYI protein family members showed an average length of 510 aa and predicted molecular weight of 57.04 kDa. Isoelectric point (pI) is an important parameter of protein that determined the net charge of a protein under certain physiological conditions. Members of MtGLYI family showed the broad difference in their pI-value ranging from 4.69 (MtGLYI-2) to 9.62 (MtGLYI-29). Out of 35 MtGLYI proteins, 24 of them showed a pI-value less than 7.0 (neutral pH) and the rest 11 showed more than 7.0. Thus both positively and negatively charged MtGLYI proteins co-exist in the system under particular physiological condition. MtGLYI proteins were predicted to be localized at cytosol, chloroplast, mitochondria, nucleus, and extracellular spaces. The majority of MtGLYI family members are found to be localized in the cytosol. Chloroplast localization of MtGLYI-21, MtGLYI-24.1, and MtGLYI-24.2 were confirmed by all three tools namely CELLO, Wolf PSORT, and ChloroP. Localization signal for mitochondria, nucleus or extra-cellular matrix were found to be predicted by either CELLO or Wolf PSORT (**Table 1**).

The length of *MtGLYII* CDS varied from 723 bp (*MtGLYII-6.2*) to 2853 bp (*GmGLYII-8.1*) with an average of 2579 bp (**Table 2**). The largest member (MtGLYII-8.1) has a polypeptide length of 950 aa with a molecular weight of 104.98 kDa; whereas MtGLYII-6.2, the smallest member has polypeptide length and molecular weight of 240 aa and 26.47 kDa, respectively (**Table 2**). In terms of pI, MtGLYII-2 and MtGLYII-6.2 showed the most acidic (4.82) and basic (8.93) value, respectively. Like MtGLYI members, the majority of MtGLYII proteins (17 out of 27) showed acidic pI-value, while rest 10 MtGLYII members showed basic pI-value (**Table 2**). Similar to MtGLYI, most of the MtGLYII proteins showed cytosolic localization. Chloroplast localization of MtGLYII-9.1 and MtGLYII-13.1 was confirmed by all three tools-CELLO, Wolf PSORT, and ChloroP; and mitochondrial localization of MtGLYII-6.1 was confirmed by both CELLO and Wolf pSORT. Nucleus and extracellular localization of few members were predicted by either CELLO or Wolf pSORT.

Like conventional glyoxalase enzymes (GLYI + GLYII), different physio-chemical properties of unique glyoxalase III proteins were analyzed. The length of *MtDJ-1* CDS was varied from 279 bp (*MtDJ-1B*) to 1356 bp (*MtDJ-1E.1*) with an average of 898 bp (**Table 3**). Consequently, the smallest MtDJ-1 member (MtDJ-1B) is 91 aa in length with a molecular weight of 10.43 kDa; while the largest one is MtDJ-1E.1 with a length of 450 aa and molecular weight of 48.20 kDa (**Table 3**). Isoelectric value (pI) varies from 5.69 (MtDJ-1D) to 9.42 (MtDJ-1E.1); with four acidic and two basic members at neutral pH. Chloroplast localization of MtDJ-1A, MtDJ-1E.1, and MtDJ-1E.2 was confirmed by all three tools-CELLO, Wolf PSORTand ChloroP; followed by cytosolic localization of MtDJ-1C and MtDJ-1D, and mitochondrial localization of MtDJ-1E.2 was confirmed by both CELLO and Wolf pSORT tools. Localization of extracellular matrix and plasma membrane of few members were only predicted by either CELLO or Wolf pSORT tool (**Table 3**).

### Chromosomal Distribution and Gene Duplication

All glyoxalase genes were found to be distributed randomly in seven different chromosomes of *Medicago* out of total eight chromosomes (**Figure 2**). A detailed chromosome map was constructed to exactly point out the newly identified *MtGLYI, MtGLYII*, and *MtDJ-1* genes on different *Medicago* chromosomes. Five of MtGLYI (*MtGLYI-25* to *MtGLYI-29*) and one of the MtGLYII genes (*MtGLYII-14*) were present in scaffold region and thus unable to point out them in chromosomes. Rest 24 *MtGLYI* genes were found to be unevenly distributed in seven *Medicago* chromosomes. Chromosome 4 contains a maximum number of eight *GLYI* genes, followed by chromosome 5 with six and chromosome 2 with four members. However, chromosomes 1 and 8 have two *GLYI* genes each, and only one *GLYI* gene each is present in chromosomes 3 and 6 (**Figure 2**). Similarly, 13 *MtGLYII* genes were found to be located on six different chromosomes (**Figure 2**). The gene density per chromosome is highly uneven, where chromosome 2 contains a maximum of five *GLYII* genes, followed by three genes in chromosome 1 and two genes in chromosome 4. However, chromosomes 3, 5, and 8 have only one *GLYII* gene each (**Figure 2**). Out of 5 *MtDJ-1* genes, maximum three genes present in chromosome 3 and one member each in chromosomes 2 and 5. All these genes were found to be distributed in a highly uneven fashion in terms of chromosome number and position. Chromosome 4 was found to be the highly dense with 10 genes in total; while chromosomes 2, 3, and 5 contain at least one member of each gene family (**Figure 2**).

Gene duplication and divergence are two most important phenomenon of plant evolution. Two major angiosperms (eudicots and monocots) are calculated to diverge from 125–140 Mya to 170–235 Mya ago (Lee et al., 2013). This largely results in subsequent chromosomal duplication and gain/loss of genes. To identify glyoxalase gene duplication among four plants (*Arabidopsis*, Rice, Soybean, and *Medicago*) genome, plant genome duplication database^12^ were analyzed (Lee et al., 2013) and the data is presented as **Table 4**. In this study, five MtGLYI and one MtGLYII sister-gene pairs have been observed (**Table 4**). The gene pairs are MtGLYI-4/MtGLYI-14, MtGLYI-5/MtGLYI-8, MtGLYI-7/MtGLYI-22, MtGLYI-12/MtGLYI-8, MtGLYI-19/MtGLYI-23, and MtGLYII-7/MtGLYII-10. Moreover, orthologous of MtGLY genes were found in three other well-characterized plant genomes of *Arabidopsis*, Soybean, and Rice. Non-synonymous to synonymous substitutions (*K*_a_/*K*_s_) ratio indicates the evolutionary history and direction of selection (Li et al., 1981; Chen et al., 2014). A pair of genes with *K*_a_/*K*_s_ < 1 indicates purifying selection, *K*_a_/*K*_s_ = 1 implies neutral drifting, and lastly *K*_a_/*K*_s_ > 1 implies positive or Darwinian selection (Juretic et al., 2005; Chen et al., 2014). *K*_a_/*K*_s_ ratio of all gene pairs was found to be below 1 indicating the influence of purifying selection in the evolution of glyoxalase genes among *Arabidopsis*, rice, soybean, and *Medicago* (**Table 4**).

### Phylogenetic Analysis of Conventional Glyoxalase Genes among Four Well-Characterized Plant Species

Glyoxalase genes from *Arabidopsis*, rice, soybean, and *Medicago* were analyzed further for their sequence similarities and evolutionary divergence through phylogenetic analysis. Three major classes GLYI family members were identified in the phylogenetic tree (**Figure 3A**). Based on various previous studies, these classes may belong to Ni^2+^-dependent GLYI, Zn^2+^-dependent GLYI, and functionally diverse inactive GLYI. Clade-I had the largest GLYI members from different plant species, while clade-II had the lowest number of members only from *Arabidopsis* and rice genome (**Figure 3A**). Clade-I contains three rice GLYI members OsGLYI-2, OsGLYI-7, and OsGLYI-11; and two members of *Arabidopsis* GLYI family AtGLYI-3 and AtGLYI-6 those have been considered as Ni^2+^-dependent GLYI enzyme (Kaur et al., 2013; Mustafiz et al., 2014). Thus, members of this clade are supposed to show Ni^2+^-dependent GLYI activity. Similarly, clade-II has previously reported Zn^2+^-dependent rice (OsGLYI-8) and *Arabidopsis* (AtGLYI-2) member which indicated to be Zn^2+^-dependent GLYI clusters. This confirmed the dominance of Ni^2+^-dependent GLYI over Zn^2+^-dependent isoform in plant species (**Figure 3A**) which had been recently proved in *Arabidopsis*, too (Jain et al., 2016). Members of clade-III are considered as functionally diverged GLYI members as it contains OsGLYI-10, which did not possess conventional GLYI activity (unpublished data).

Similarly, phylogenetic analysis of GLYII proteins could be divided into three major classes (I–III) (**Figure 3B**). One of rice GLYII protein (OsGLYII-1) and one *Arabidopsis* GLYII protein (AtGLYII-3) was previously reported to possess sulfur dioxygenase (SDO) activity (Kaur et al., 2014b). Both of them were present in the clade-III, representing the functional divergence members of GLYII family. As AtGLYII-2 was found to be localized in mitochondria (Marasinghe et al., 2005), other members of clade II (one from rice, OsGLYII-2), four proteins from soybean and one from *Medicago*, MtGLYII-12) could be mitochondrial localized. Lastly, Clade-I might contain all the active and cytoplasmic localized GLYII members from four plant species.

### Structural and Sequence Analysis of MtGLYI Proteins

**FIGURE 5:**
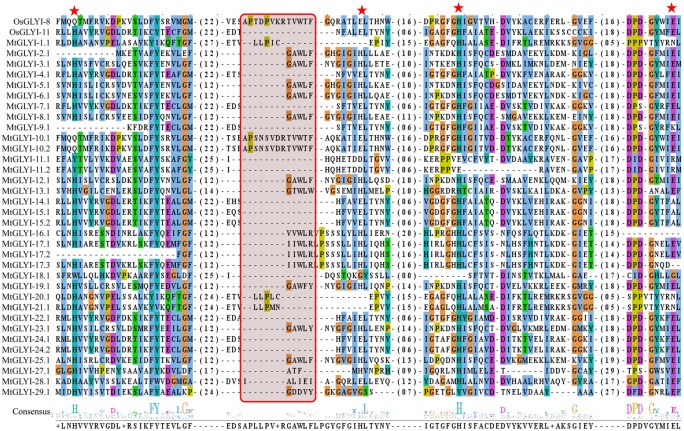
**Sequence alignment of GLYI domains of all MtGLYI proteins along with that of OsGLYI-11.2 and OsGLYI-8.** Conserved GLYI domains (first one in case of two domain) of all MtGLYI proteins were aligned with that of previously reported Ni^2+^-dependent OsGLYI-11.2 and Zn^2+^-dependent OsGLYI-8 using ClustalW program. The picture was generated using Jalview multiple alignment editor programs. Four conserved metal binding sites of GLYI proteins were represented as red stars, while region specific for Zn^2+^-dependent isoform was marked with a red shaded box.

The presence of conserved glyoxalase domain (PF00903) in all the predicted 35 MtGLYI proteins was analyzed using Pfam. Analyses revealed that all MtGLYI protein has at least one GLYI domain, while some of them have two. Out of 35, nine MtGLYI proteins contained two consecutive GLYI domains (**Figure 4**). Single GLYI protein with two domains had been reported from *G. max* (Ghosh and Islam, 2016), *S. cerevisiae* (Frickel et al., 2001), *O. sativa* (Mustafiz et al., 2014), and *Plasmodium falciparum* (Deponte et al., 2007). Apart from conserved GLYI domain, some proteins have addition domains, such as WD domain (PF00400), Alpha/beta hydrolase family (PF12697) and Enoyl-CoA hydratase/isomerase (PF00378). The role of these domains in association with GLYI proteins is still not clear. GLYI domain (only N-terminal one in a case of two domain containing members) sequences of all MtGLYI proteins were aligned with that of Ni^2+^-dependent OsGLYI-11.2 and Zn^2+^-dependent OsGLYI-8 (Kaur et al., 2013) proteins to predict on their metal ion dependency and catalytic activity (**Figure 5**). Enzymatic activity of GLYI proteins has been found to be dependent on metal ions (Kaur et al., 2013). This dependency could be easily predicted based on the size of GLYI domain; domain length of ∼120 aa showed Ni^2+^-dependence and length of 142 aa showed Zn^2+^-dependence (Kaur et al., 2013). According to the alignment, Zn^2+^-dependent OsGLYI-8 has an extra stretch of 12 aa (**Figure 5**, marked with rectangle), that is completely absent in Ni^2+^-dependent OsGLYI-11.2 proteins. Proteins with this stretch of amino acids such as MtGLYI-10.1 and MtGLYI-10.2 are found to be Zn^2+^ dependent, while proteins like MtGLYI-4, MtGLYI-7, MtGLYI-9, MtGLYI-11, MtGLYI-14, MtGLYI-15, MtGLYI-18, MtGLYI-22, and MtGLYI-24 might be Ni^2+^ dependent. Some of the proteins possess partial regions, thus difficult to predict (**Figure 5**).

The active site of GLYI proteins had a conserved motif of H/QEH/QE irrespective of their metal ion dependency. These four residues were found to be conserved in both OsGLYI-8 and OsGLYI-11.2 proteins, and marked with red stars (**Figure 5**). Among these four residues, the position of first glutamate residue showed maximum divergence in GmGLYI proteins. MtGLYI-11.1 and MtGLYI-11.2 proteins showed variation in all the four conserved active side residues. Proteins with the presence of all these four highly conserved residues are expected to have functional GLYI enzyme activity as per suggestion of previous studies (Mustafiz et al., 2014; Ghosh and Islam, 2016). Eight MtGLYI proteins such as MtGLYI-4.1, MtGLYI-7.1, MtGLYI-10.1, MtGLYI-10.2, MtGLYI-22.1, MtGLYI-24.1, MtGLYI-24.2, and MtGLYI-28.1 possess all four residues and expected to have function GLYI enzyme activity.

### Sequence Analysis of MtGLYII Proteins for Conserved Domain and Active Sites

**FIGURE 6:**
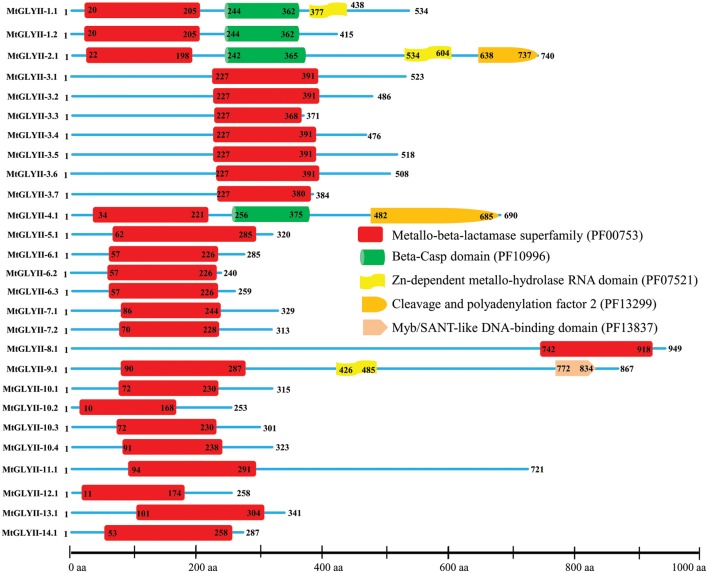
**Schematic representation of MtGLYII proteins structure.** Protein structures of 27 MtGLYII proteins are shown along with the names of all members on the left side of the figure. MtGLYII proteins were analyzed using Pfam (http://www.sanger.ac.uk/Software/Pfam) for the presence of functional domain(s). All these proteins possess conserved Metallo-beta-lactamase superfamily (PF00753) along with some other domains such as Beta-Casp domain (PF10996), Zn-dependent metallohydrolase RNA domain (PF07521), Cleavage and polyadenylation factor 2 (PF13299), and Myb/SANT-like DNA-binding domain (PF13837). Different domains are marked by different colored artworks denoted at the right side of the figure. The exact position of each domain or full-length proteins is indicated by the exact amino acid number inside or outside the box. The relative position of the domains could be interpreted by the scale given below.

**FIGURE 7:**
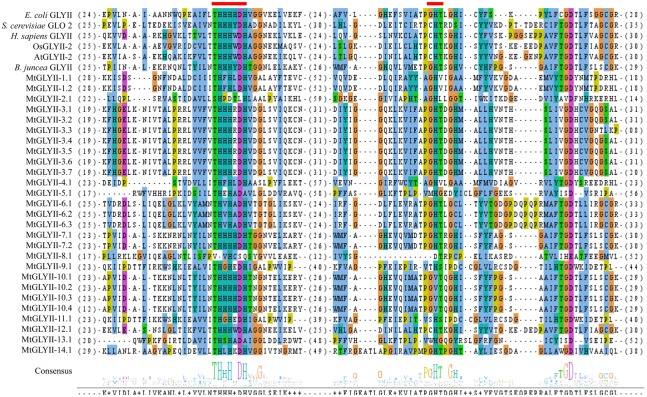
**Sequence alignment of GLYII domains of all MtGLYII proteins along with that of other reported GLYII proteins from various species.** Conserved GLYII domains of all MtGLYII proteins were aligned with that of previously reported GLYII proteins from *E. coli*, *S. cerevisiae*, *H. sapiens*, rice (OsGLYII-2), *Arabidopsis* (AtGLYII-2), and *B. juncea* using ClustalW program. The picture was generated using Jalview multiple alignment editor programs. Two conserved metal binding sites of GLYII proteins were indicated as red boxes.

Pfam analysis confirmed the presence of conserved Metallo-beta-lactamase domain (PF00753) in all MtGLYII proteins either alone or with some other domains (**Figure 6**). Out of total 27 proteins, 22 MtGLYII proteins had only Metallo-beta-lactamase domain, while rest 5 contained additional domain such as Beta-Casp domain (PF10996), Zn-dependent metallo-hydrolase RNA domain (PF07521), Cleavage and polyadenylation factor 2 (PF13299), Myb/SANT-like DNA-binding domain (PF13837) (**Figure 6**). The significance of these additional domains is not clear in relation to GLYII enzyme activity. Similar to GLYI, GLYII proteins contained two well-conserved domain; one is metal binding motif (THXHXDH) and another one is active site motif (C/GHT) (Ghosh et al., 2014). The presence of both these motifs is crucial for a functional GLYII enzyme. Therefore, sequences of all MtGLYII proteins were aligned with that of *E. coli*, yeast, human, rice, *Arabidopsis* and *Brassica* GLYII proteins (**Figure 7**). The position of both these motifs was marked with red boxes (**Figure 7**). Out of 27 identified MtGLYII proteins, only two (MtGLYII-2.1 and MtGLYII-8.1) do not possess the conserved metal binding residues, but 13 of them do not have the active site motif (**Figure 7**). MtGLYII-1.1, MtGLYII-2.1, MtGLYII-2.2, MtGLYII-4.1, MtGLYII-5.1, MtGLYII-8.1, MtGLYII-9.1, MtGLYII-10.1-4, MtGLYII-11.1, and MtGLYII-13.1 proteins are either missing metal binding or active side residues, thus might not possess functional GLYII enzyme activity.

### Analysis of Unique Glyoxalase III Proteins in *Medicago*

**FIGURE 8:**
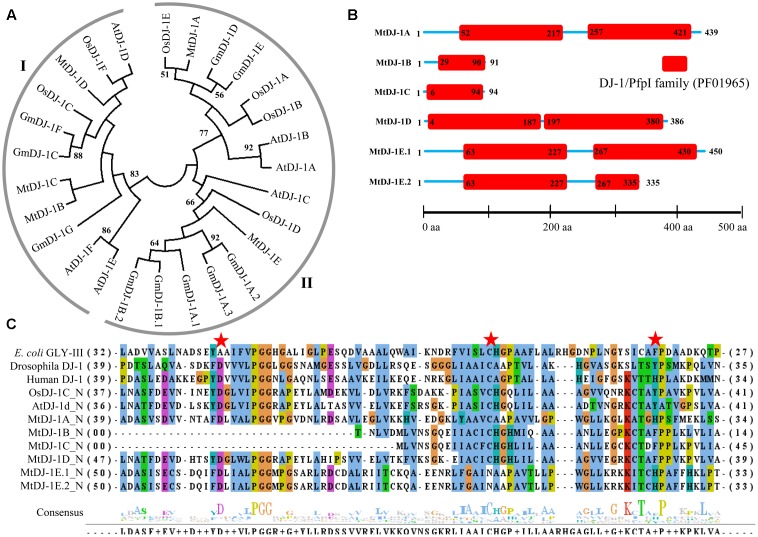
**Structural and sequence analysis of MtDJ-1 proteins.** **(A)** Phylogenetic analysis of DJ-1 protein family members from *Medicago*, *Arabidopsis*, rice, and soybean was performed using their full-length amino acid sequences and neighbor-joining method with 1000 bootstrap value. **(B)** Protein structures of 6 MtDJ-1 proteins were analyzed using Pfam (http://www.sanger.ac.uk/Software/Pfam) for the presence of functional DJ-1/PfpI domain (PF01965). The exact position of each domain is indicated by the exact amino acid number inside the red box, while the amino acid number of full length of protein is indicated at each end of the line. The relative position of the domains could be interpreted by the scale given below. **(C)** Conserved DJ-1/PfpI domain (N-terminal one in case of two) of all MtDJ-1 proteins were aligned with that of previously reported well-characterized novel GLYIII proteins of *E. coli*, *D. melanogaster*, *H. sapiens*, rice (OsDJ-1C), and *Arabidopsis* (AtDJ-1d) using ClustalW program. Jalview multiple alignment editor programswere employed to generate the picture. Three conserved active side residues are marked with red stars.

The presence of unique glyoxalase III proteins has been reported in many microorganisms and plants. GLYIII activity was found in the proteins with DJ-1/PfpI domain. Like conventional glyoxalase enzymes, DJ-1 proteins from *Arabidopsis*, rice, soybean, and *Medicago* were analyzed using Mega 5.2 tool (**Figure 8A**). There were two major clades in the tree, named- clades I and II. Among them, AtDJ-1D and OsDJ-1C were experimentally proven to have substantial GLYIII enzymatic activity (Kwon et al., 2013; Ghosh et al., 2016). Proteins present in clade-I along with these two might have significant GLYIII activity; while clade-II members could have less activity.

All previously reported rice and *Arabidopsis* DJ-1 proteins were found to contain two DJ-1/PfpI domains, while that of *E. coli*, *Drosophila*, and human were found to contain only single domain (Ghosh et al., 2016). DJ-1/PfpI domain has an average size of around 140 to 150 amino acids in all species except *E. coli*. Sequence analysis of MtDJ-1 proteins revealed that most of them have two DJ-1/PfpI domain, too (**Figure 8B**). Only MtDJ-1B and MtDJ-1C has single DJ-1/PfpI domain with a size of 69 and 88 amino acids each. These two might have partial DJ-1/PfpI domain. However, the domains of other MtDJ-1 proteins are connected by a linker of 40 amino acids (MtDJ-1A and MtDJ-1E) or 10 amino acids (MtDJ-1D). The N-terminal DJ-1/PfpI domains of all MtDJ-1 proteins were aligned with that of *E. coli*, *Drosophila*, human DJ-1 proteins; and AtDJ-1D and OsDJ-1C proteins (**Figure 8C**). All reported DJ-1 proteins with GLYIII contain a conserved catalytic triad of aspartate/glutamate, cysteine, and tyrosine/histidine residues (Ghosh et al., 2016). All these three residues were marked with red stars (**Figure 8C**). Among them, the central cysteine residue is the key for GLYIII enzymatic activity due to it’s directly involvement in catalysis (Lin et al., 2012). All MtDJ-1 proteins possessed this highly conserved cysteine residues, except MtDJ-1E (**Figure 8C**). The absence of this important residue in MtDJ-1E protein might lead to complete lack of GLYIII activity as proven by previously for AtDJ-1C (Kwon et al., 2013). However, the absence of any of other two conserved residues led to partial loss of GLY III activity in case of AtDJ-1e and AtDJ-1f (Kwon et al., 2013). Thus, MtDJ-1A should be the most efficient GLYIII enzyme of *Medicago* with all three conserved residues, followed by MtDJ-1D (**Figure 8C**).

### Expression Profiling of Glyoxalase Genes in *M. truncatula*

**FIGURE 9:**
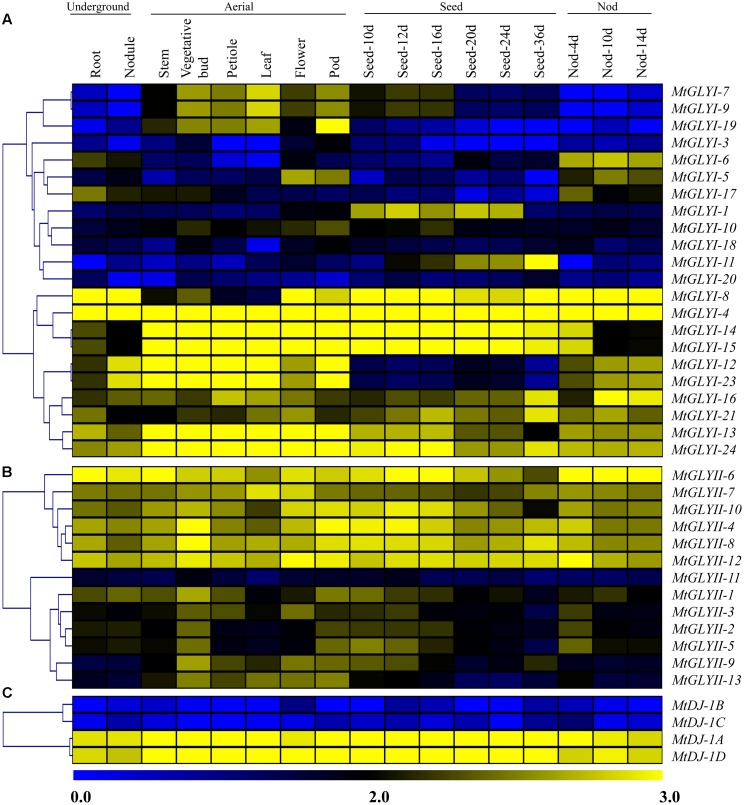
**Expression profiling of *Medicago* glyoxalase genes with hierarchical clustering at different developmental stages/tissues.** Genome-wide microarray data of conventional GLYI **(A)** and GLYII **(B)**; and novel glyoxalase III **(C)** genes were obtained from the *Medicago truncatula* gene expression Atlas (MtGEA) Project database (https://mtgea.noble.org/v2/). Normalized transcript data was obtained for 17 different tissues, including underground tissues-root and nodule; aerial tissues- stem, vegetative bud, petiole, leaf, flower, pod; seed development (seed of 10, 12, 16, 20, 24, and 36 days); and nod development (nod of 4, 10, and 14 days). These normalized expression data was used to generate heatmap with hierarchical clustering based on the Manhattan correlation with average linkage using MeV software package. Color scale below heat map shows the level of expression; yellow indicates high transcript abundance while blue indicates a low level of transcript abundance.

To investigate the expression patterns of *Medicago* glyoxalase genes, expression data for a diverse set of 17 *Medicago* tissues was retrieved and analyzed. All these tissues could be broadly divided into four classes; such as underground, aerial, seed and nod; and represented as heat maps (**Figures 9A–C**). Different members of *MtGLY* gene families showed different tissue specificity. Among all *MtGLYI* genes, *MtGLYI*-4 showed the highest level of constitutive expression in all these analyzed tissues, followed by *MtGLYI*-24, *MtGLYI*-13, *MtGLYI*-21, and *MtGLYI*-16. *MtGLYI*-8 also maintained a high level of expression except for some aerial tissues; while *MtGLYI*-14 and *MtGLYI*-15 showed low expression only in underground and nod tissues. There are some clusters of genes that showed expression in particular type of tissues only. *MtGLYI*-7, *MtGLYI*-9 and *MtGLYI*-19 showed medium to high-level expression in aerial tissues only, while *MtGLYI*-5, *MtGLYI*-6 and *MtGLYI*-17 showed a similar pattern of expression only in underground and nod tissues. The presence of seed-specific *GLYI* transcripts has been previously reported in rice, *Arabidopsis*, and soybean (Mustafiz et al., 2014; Ghosh and Islam, 2016). Similarly, *MtGLYI*-1 and *MtGLYI*-11 has medium to high level of expression in different seed maturation stages and low expression in other tissues (**Figure 9A**). However, some members maintained the high level of expression in all tissues except for seed only, such as *MtGLYI-12* and *MtGLYI-23*. This indicates toward the presence of a complex regulatory network for *MtGLYI* gene expression.

On the other hand, two distinct clades were observed in case of *MtGLYII* and *MtDJ-1* gene expression data (**Figures 9B,C**). One set of genes maintained high level of constitutive expression in all tissues, while the other set of genes had very low level of expression. Out of 13 analyzed *MtGLYII* genes, 6 genes such as *MtGLYII*-4, *MtGLYII*-6, *MtGLYII*-7, *MtGLYII*-8, *MtGLYII*-10, and *MtGLYII*-12 showed medium to high-level expression in all the tissues with very few exceptions (**Figure 9B**). Among others, *MtGLYII*-11 showed the lowest level of expression in all the tissues, while *MtGLYII*-1, *MtGLYII*-3, *MtGLYII*-9, and *MtGLYII*-13 genes showed significant expression in the aerial tissues only. In the case of *MtDJ-1* family members; *MtDJ-1A* and *MtDJ-1D* showed a high level of expression in all these tissues, while *MtDJ-1B* and *MtDJ-1C* showed the lowest level (**Figure 9C**). As compared to *MtGLYI* family members, *MtGLYII* and *MtDJ-1* family members showed less complexity and tissue specificity.

### Expression of *MtGLYI*, *MtGLYII*, and *MtDJ-1* Genes in Response to Salinity and Drought

**FIGURE 10:**
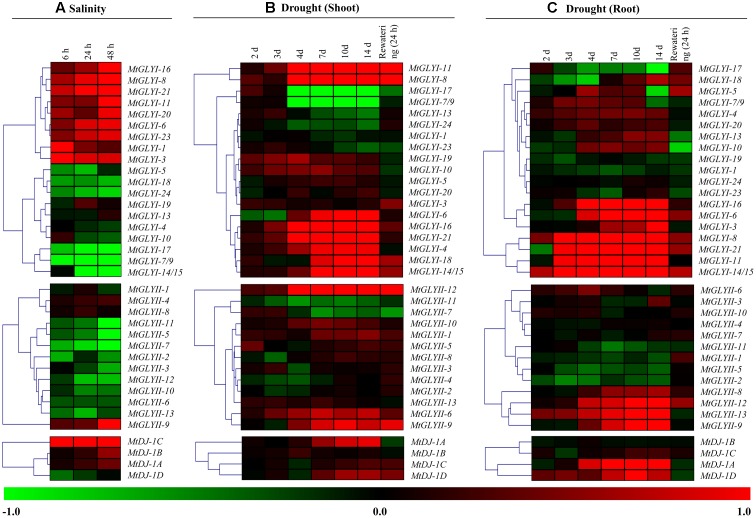
**Expression analyses of *Medicago* glyoxalase genes in response to two devastating abiotic stresses.** Normalized and curated perturbation expression data of the *Medicago* glyoxalase genes were retrieved from publicly available Genevestigator Affymetrix Medicago genome array (https://www.genevestigator.com/gv/plant.jsp) using the already identified corresponding probesets of experiments with id: MT-00011 and MT-00013 (Details provided in the “Materials and Methods” section). Relative expression data of all available *MtGLYI*, *MtGLYII*, and *MtDJ-1* were combined and analyzed in response to different duration of salinity **(A)**; and drought with shoot tissue **(B)** and root tissue **(C)**. Fold change of expression data was calculated by comparing with the corresponding mock samples. Heat maps with hierarchical clustering were generated using Manhattan distance metric method of MeV software package. Color scale below heat map shows the type of transcript alteration; red indicates up-regulation and green indicate down-regulation of the corresponding transcript.

To investigate the physiological role of *Medicago* glyoxalase genes in response to abiotic stress, corresponding transcript expression was analyzed under salinity and drought stresses. In response to salt stress (3 days old Jemalong A17 seedlings were treated with 180 mM NaCl) (Li et al., 2009), log_2_ fold change ratio was analyzed at 6, 24, and 48 h of stress as compared to their 0 h expression level. A cluster of *MtGLYI* genes-*MtGLYI-16*, *MtGLYI-8*, *MtGLYI-21*, *MtGLYI-11*, *MtGLYI-20*, *MtGLYI-6*, *MtGLYI-23*, *MtGLYI-1*, and *MtGLYI-3* showed early up-regulation (6 h) with further enhancement with stress duration (48 h). However, another set of *MtGLYI* genes-*MtGLYI-17, MtGLYI-7/9, MtGLYI-14/15, MtGLYI-5, MtGLYI-18*, and *MtGLYI-24* showed strong down-regulation at all three-time points (**Figure 10A**). Rest members-MtGLYI-4, MtGLYI-10, MtGLYI-13, and MtGLYI-19 showed slight alteration as compared to the untreated control sample. On contrary, only one MtGLYII member-*MtGLYII-9* showed strong up-regulation, followed by *MtGLYII-4* and *MtGLYII-8* (**Figure 10A**). Similarly, *MtDJ-1C* showed stable up-regulation from 6 to 48 h of salt stress, followed by *MtDJ-1B* and *MtDJ-1A* (**Figure 10A**). Rest of MtGLYII and MtDJ-1 members showed a different degree of down-regulation.

A complex differential expression pattern was observed for all three gene families in response to drought stress at both shoot (**Figure 10B**) and root (**Figure 10C**). Most of the genes showed less sensitivity during early days of stress (2 and 3 days). From day-4 and onward, some *MtGLYI* genes (*MtGLYI-11, MtGLYI-8, MtGLYI-6, MtGLYI-16, MtGLYI-21, MtGLYI-4, MtGLYI-18*, and *MtGLYI-14/15*) showed strong up-regulation; while two other members (*MtGLYI-17* and *MtGLYI-7/9*) showed drastic down-regulation. In case of MtGLYII; *MtGLYII-12, MtGLYII-6*, and *MtGLYII-9* showed a similar pattern of up-regulation from 4 to 14 days of drought stress. All these up/down-regulated genes maintain this pattern after 24 h of rewatering, too. Similarly, drought stress causes severe alteration of *MtGLYI, MtGLYII, and MtDJ-1* genes at root tissues (**Figure 10C**). *MtGLYI-16, MtGLYI-6, MtGLYI-3, MtGLYI-8, MtGLYI-21, MtGLYI-11*, and *MtGLYI-14/15* genes showed sharp up-regulation from day-3 to onward at root tissue, and come down to normal level after rewatering (**Figure 10C**). Likewise, *MtGLYII-12, MtGLYII-13, MtGLYII-9*, and *MtDJ-1A, MtDJ-1D* maintained up-regulated transcripts among *MtGLYII* and *MtDJ-1* families from very early to late drought stress. As root has a direct contact with soil water, re-watering brings back the expression level of most glyoxalase genes into their ground level at root tissue (**Figure 10C**). All these expression data indicated the diverse role of different conventional and unique glyoxalase members in different stress modulation pathways of *Medicago* plant.

## Discussion

Previous studies have shown that glyoxalase proteins play important role in the development and abiotic stress modulation of a plant (Kaur et al., 2014a,c). Genome-wide identification of glyoxalase gene family has been carried out in *Arabidopsis*, rice, and soybean (Mustafiz et al., 2011; Ghosh and Islam, 2016). The present study of genome-wide analysis of glyoxalase genes in *M. truncatula* identified a total of 29 GLYI, 14 GLYII, and 5 DJ-1 genes. The number of glyoxalase genes in *M. truncatula* was found to be the maximum as compared to the previous reports; *Arabidopsis* (10 GLYI, 5 GLYII, and 6 DJ-1 genes) (Mustafiz et al., 2011; Kwon et al., 2013), rice (11 GLYI, 3 GLYII, and 6 DJ-1 genes) (Mustafiz et al., 2011; Ghosh et al., 2016) and soybean (24 GLYI, 12 GLYII, and 7 DJ-1 genes) (Ghosh and Islam, 2016) (unpublished data). The number of glyoxalase genes in four different plant species could not be correlated with their respective genome size. The genome size of *Medicago* is 375 Mb (Young et al., 2011); soybean genome size is 1.1 Gb (Schmutz et al., 2010); size of rice genome is 466 Mb (Yu et al., 2002) and that of *Arabidopsis* is 125 Mb (Bennett et al., 2003). In spite of one third genome size as compared to soybean, another legume plant *Medicago* has a greater number of conventional glyoxalase genes and an almost similar number of unique glyoxalase genes (**Tables 1**–**3**). However, a similar level of both conventional and unique glyoxalase genes is present in *Arabidopsis* and rice even though rice has four times bigger genome size than *Arabidopsis*. Plants tend to duplicate its important genes to generate novel isoform or increase number to adopt with different adverse conditions and developmental process (Gu et al., 2003; Chen et al., 2014). In the present analysis, a total of six duplicated glyoxalase gene pairs were observed in *Medicago* (**Table 4**). Moreover, orthologs of *Medicago* glyoxalase genes were found in *Arabidopsis*, rice, and soybean too. Maximum 11 orthologs pairs were found with *Arabidopsis*, followed by nine with soybean and five with rice (**Table 4**).

One of the possible reasons behind the greater number and gene duplication event of plant glyoxalase genes as compared to their animal counterpart is the functional divergence of genes (Gu, 2003). Different types of subfunctionalization or neofunctionalization have arrived due to the functional divergence (Gu, 2003). In the present study, out of 35 MtGLYI proteins several of them did not possess all four conserved functional metal binding site and might be non-functional GLYI enzyme (**Figure 5**). Glutathione *S*-transferase activity has been observed for one of the earlier predicted soybean GLYI (accession no. X68819) (Skipsey et al., 2000), while sulfur dioxygenase-like activity observed for *Arabidopsis* (AtGlx2-5) and rice (OsGLYII-1) GLYII proteins (Kaur et al., 2014b). Moreover, GLYI proteins could be broadly divided into two classes based on their metal activation; Zn^2+^ and non-Zn^2+^ (mainly Ni^2+^/Co^2+^) (Jain et al., 2016). This metal ion specificity could be easily predicted based on their GLYI domain’s amino acid sequence and length (Suttisansanee et al., 2011; Kaur et al., 2013). Based on these criteria, maximum MtGLYI proteins were predicted be Ni^2+^/Co^2+^-activated (**Figure 5**) similar to previously predicted functional GLYI of soybean (16 out of 20) (Ghosh and Islam, 2016), rice (3 out of 4 active OsGLYIs) (Kaur et al., 2013) and *Arabidopsis* (2 out of 3 functional AtGLYIs) (Jain et al., 2016). Coexistence of both these metal activated form has been reported recently in model plant *A. thaliana* (Jain et al., 2016), and earlier in a eubacterial species, *Pseudomonas aeruginosa* (Sukdeo and Honek, 2007).

Glyoxalase transcripts were found to be altered in response to certain tissue or developmental stages specificity in the previous studies on *Arabidopsis*, rice, and soybean (Mustafiz et al., 2011; Ghosh and Islam, 2016). Transcript abundance analysis of glyoxalase genes at different developmental stages revealed the constitutive expression pattern of *AtGLYI*2, *AtGLYI*3, *AtGLYI*6, *AtGLYI*9, *AtGLYI*11, *AtGLYII*2, and *AtGLYII*5 of *Arabidopsis*; and *OsGLYI*2, *OsGLYI*4, *OsGLYI*6, *OsGLYI*7, *OsGLYI*8, *OsGLYI*11, *OsGLYII*2, and *OsGLYII*3 at all the stages (Mustafiz et al., 2011). Similar pattern of constitute expression was observed for *GmGLYI*7, *GmGLYI*8, *GmGLYI*21, and *GmGLYII*8 transcripts of soybean at the developmental stages (Ghosh and Islam, 2016). However, some other glyoxalase members such as *AtGLYI*8, *OsGLYI*3, and *OsGLYI*10 showed highly tissue specific (seed) expression (Mustafiz et al., 2011). Expression profile of all the conventional glyoxalase (*MtGLYI* and *MtGLYII*) and novel glyoxalase (*MtDJ-1*) genes was analyzed at different developmental stages (**Figure 9**). *Medicago* glyoxalase transcripts showed a similar pattern of tissue preferences. Out of all members; *MtGLYI*-4, *MtGLYII*-6, *MtDJ-1*A, and *MtDJ-1*D showed the constitutive high level of expression at all the analyzed stages. Members of all three gene families (*MtGLYI, MtGLYII*, and *MtDJ-1*) showed distinct two clusters according to their transcript abundance in underground, aerial, seed, and pod tissues (**Figure 9**). One cluster of genes maintained high-level expression in all analyzed tissues, while the other cluster showed a low level of expression. Meanwhile, some other members such as *MtGLYI*-1, *MtGLYI*-6, *MtGLYI*-7, *MtGLYI*-9, *MtGLYI*-13, *MtGLYII*-1, and *MtGLYII*-13 showed preference toward certain stages/tissue types, indicated their imperative role in the developmental transition/regulation. Genes with low transcript abundance in the developmental stages/tissues might have other stress or cellular or metabolic regulation.

Various abiotic stresses cause increased *in vitro* accumulation of MG in plant tissues (Kaur et al., 2014c). The role of MG detoxification glyoxalase machinery had been well characterized in literature that provided tolerance against multiple abiotic stresses (Graham and Vance, 2003; Yu et al., 2006). Recently, manipulation of rice glyoxalase pathway has been reported to provide tolerance against three major abiotic stresses such as salinity, drought and heat; and to reduce sheath blight fungus (*Rhizoctonia solani*) induced damage (Gupta et al., 2017). The possible mechanisms behind these observed tolerance against diverse abiotic and biotic stresses might involve improved MG detoxification, reduced oxidative damage, protected ultra-structure of organelles (chloroplast and mitochondria), and maintained photosynthetic machinery (Gupta et al., 2017). Similar pattern of stress tolerance was observed when the activity of complete glyoxalase pathway was enhanced in transgenic tobacco (Singla-Pareek et al., 2003) and tomato plant (Alvarez Viveros et al., 2013). However, over-expression of either GLYI or GLYII gene also provides tolerance against various abiotic stresses (Singla-Pareek et al., 2008; Ghosh et al., 2014; Mustafiz et al., 2014; Kaur et al., 2017). Thus, the stress tolerance potential of glyoxalase members have been well proved by various studies.

Different glyoxalase members of *Arabidopsis* and rice such as *AtGLYI*4, *AtGLYI*7, *OsGLYI*6, and *OsGLYI*11 were previously reported for their highly stress inducible expression pattern (Mustafiz et al., 2011). Similarly, several soybean glyoxalase members such as *GmGLYI*6, *GmGLYI*9, *GmGLYI*20, *GmGLYII*5, and *GmGLYII*10 were reported for their highly abiotic stress responsive expression (Ghosh and Islam, 2016). Moreover, few other soybean glyoxalase members such as *GmGLYI*-6/9, *GmGLYI*-20, *GmGLYII*-6, and *GmGLYII*-10 showed biotic stress specific modulation in response to various pathogenic infection (Ghosh, 2016). Stress specific transcript alteration is a highly complex process and vary depending on the duration and type of stress; and genotype of plant. Expression pattern of all *Medicago* glyoxalase genes was further analyzed in response to two devastating abiotic stresses-salinity and drought (**Figure 10**). Different members of *Medicago* glyoxalase gene families responded differently in response to the duration of stress (salinity and drought) and types of tissue analyzed (drought) (**Figure 10**). *MtGLYI*-8, *MtGLYI*-21, *MtGLYII*-9, and *MtDJ-1*C found to be the highly up-regulated member in response to salinity; while *MtGLYI*-8, *MtGLYI*-11, *MtGLYI*-21, *MtGLYII*-9, *MtGLYII*-13, and *MtDJ-1*A are the drought specific up-regulated members (**Figure 10**). Thus *MtGLYI*-8, *MtGLYI*-21, and *MtGLYII*-9 found to be the highly up-regulated abiotic stress responsive member that could be characterized further. Overall the present study provides basic information about *Medicago* glyoxalase members that will facilitate the identification of appropriate candidate gene(s) for generating abiotic stress resistant crop plants.

## Conclusion

In summary, a comprehensive *in silico* genome-wide analysis of *Medicago* glyoxalase gene families (*MtGLYI, MtGLYII*, and *MtDJ-1*) were performed. A total of 29 *MtGLYI*, 14 *MtGLYII* and 5 unique GLYIII (*MtDJ-1*) genes were identified from *M. truncatula* genome sequence that code for 35 GLYI, 27 GLYII, and 6 DJ-1 proteins, respectively. This is the largest identified glyoxalase gene family till date in any organism. All the identified members were further investigated for their classification, evolution, metal dependency, putative enzyme activity, and tissue/developmental-specific expression. Transcript abundance data of *Medicago* glyoxalase genes showed their major participation in the regulation of *Medicago* development. Specifically, the present study identified few *Medicago* glyoxalase members could be explored further their stress tolerance potential. In future, functional analyses of these genes will enable them as an ideal candidate for transgenic applications.

## Author Contributions

AG designed and performed the experiments, analyzed the data and wrote the manuscript.

## Conflict of Interest Statement

The author declares that the research was conducted in the absence of any commercial or financial relationships that could be construed as a potential conflict of interest.

## Footnotes

1 http://www.jcvi.org/medicago/

2 http://pfam.xfam.org/

3 http://cello.life.nctu.edu.tw/

4 http://www.genscript.com/wolf-psort.html

5 http://www.cbs.dtu.dk/services/ChloroP/

6 http://chibba.agtec.uga.edu/duplication/index/downloads

7 https://mtgea.noble.org/v2/

8 http://www.affymetrix.com/analysis/index.affx?navMode=cat530006\&aId=netaffxNav

9 http://mev.tm4.org

10 https://genevestigator.com/gv/doc/intro_plant.jsp

11 http://www.ncbi.nlm.nih.gov/geo/query/acc.cgi?acc=GSE14029

12 http://chibba.agtec.uga.edu/duplication/index/downloads

## Supplementary Material

**FILE S1 | Sequences of glyoxalase I proteins from various species used for phylogenetic analysis of MtGLYI proteins.**

**FILE S2 | Sequences of glyoxalase II proteins from various species used for phylogenetic analysis of MtGLYII proteins.**

**FILE S3 | Sequences of novel glyoxalase III (DJ-1) proteins from various species used for phylogenetic analysis of MtDJ-1 proteins.**

**FILE S4 | Details information about the gene expression experiments.**

## Abbreviations

aa: amino acid
bp: base pair
d: day
GLYI: glyoxalase I
GLYII: glyoxalase II
GSH: reduced glutathione
h: hour
MG: methylglyoxal
