# Supplementary material for: Genome-Wide Identification of Glyoxalase Genes in Medicago truncatula and Their Expression Profiling in Response to Various Developmental and Environmental Stimuli
Source: Front Plant Sci. 2017 Jun 1;8:836. doi: 10.3389/fpls.2017.00836 (PMC5452422; doi:10.3389/fpls.2017.00836)
Supplement: Supplementary file 1 [file Data_Sheet_1.DOCX]

>OsGLYI-1

MVNTTAGVKCGGGGAALPLSTLNHVSLVCRSLSTSLTFYRDFLGFVSVRRPGSFDFDGAWLFNYGIGIHLLQAEDPESMPPNKEINPKDNHISFTCESMEAVQRRLKEMGVRYVQRRVEEGGVYVDQIFFHDPDGFMIEICTCDKLPVVPLDAAAAHSIFAGRSPPPPVACKIRPVKQPSATKLGSVAAGGCVGEVIVVDAINGAAAAGGGGAMS

>OsglyI2

MRALPMAAGRAAAVAACASPAVPRRSLLLSTAAAGEPPCRPPADSSSPSKFSRFDRSAVRLLGWTAALQPEPVRLTRGASAAPKLRASPPDAAQAAAAFGSKEEAFAWAKSDNRRLLHVVYRVGDIDRTIKFYTECLGMKLLRKRDIPEEKYTNAFLGYGAEDNHFVVELTYNYGVDKYDIGAGFGHFGIAVDDVAKTVELIRAKGGKVTREPGPVKGGKTVIAFVEDPDGYKFEILERPGTPEPLCQVMLRVGNLDRAISFYEKACGMELLRKRDNPEYKYTVAMMGYGPEDKNAVLELTYNYGVTEYDKGNAYAQIAIGTDDVYKTAEVVKLFGGQVVREPGPLPGINTKITSILDPDGWKSVFVDNIDFAKELE

>OsglyI3

MTMTMSNEQGKPEANVRGGRRSGHRVHARHLPGLPTLAAAPARTNGSSWAPGKTEHCTAQHKRRLQAVRDKPQQASVMASEGAVSPAFAYTVVYVKDVAKSAAFYSAAFGYTVRRLDQSHKWAELESGTTTIAFTPLHQRETDALTGAVQLPDSAGERGPVEICFDYADVDAAYRRAVDSGAVPVSPPEQKSWGQKVGYVRDIDGIIVRMGSHVRA

>OsglyI4

MATLQLNHVARETDDVRRLAAFYEEVLGFERVASPNYPAFQVAWLRLPGTPGVALHIIERDPAAAPAAVAPGAAGAPPAQLPRRHHLAFSVADYDGFLTGLKARGTDVFEKTQPDGRTRQVFFFDPDGNGLEVTSSGTGDM

>OsGLYI-5

MGSEAPDPAVAASVPLVRLNHVSFQCTSVEKSVDFYRRVLGFELIKRPESLNFNGAWLYKYGMGIHLLQRGDDADGCSIPTRPLPAINPMGNHVSFQCSDMAVMKARLRAMDREFVVRKVWDGETVVDQLFFHDPDGNMIEVCNCENLPVIPLIVASTPGLPELLPPAMQTNVHG

>OsglyI6

MVNTAAVAAAKGSRGSGLPLASLNHISIVCRSLQESLTFYTDVLGFFPVRRPGSFDFDGAWLFNYGIGIHLLQAEDPDSLPGKTEINPKDNHISFQCESMVAVERRLKELGIPYIQRCVEEGGIYVDQIFFHDPDGFMIEICNCDNLPVVPLGADQPLVMAACKRAAVIKQQQQASSSPATAAAAAQCAVPSSTKAIHVGEEAHISCA

>OsglyI7

MARLLLPLPIAAAAASRLRLPVLSSSVARREALLFGGRVAAARAPVRLARRGVSAGAEAGGSSSAAAAAQVIGQDEAVEWVKKDRRRMLHVVYRVGDLDKTIKFYTECLGMKLLRKRDIPEERYTNAFLGYGPEDSHFVVELTYNYGVESYDIGTAFGHFGIAVEDVAKTVDLIKAKGGTVTREPGPVKGGKSVIAFIEDPDGYKFELIERGPTPEPLCQVMLRVGDLDHAINFYEKAFGMELLRKRDNPQYKYTIAMMGYGPEDKNAVLELTYNYGVKEYDKGNAYAQIAISTDDVYKTAEVIRQNGGQITREPGPLPGINTKITACTDPDGWKTVFVDNVDFLKELEE

>OsglyI8

MAAAAIAAASLLPSSAFALRRLSSAANVSRFAQLKRFDRARRFAPAAAMSTSSGPKEAPANNPGLQAPSEKDPATKGYFMQQTMFRVKDPKVSLDFYSRVMGMSLLKRLDFPEMKFSLYFLGYEDVESAPTDPVKRTVWTFGQRATLELTHNWGTENDPEFKGYHNGNSDPRGFGHIGVTVHDVYKACERFERLGVEFVKKPDDGKMKGIAFIKDPDGYWIEIFDLNRIGAVTAEAS

>OsGLYI-9

MAARCLSSLALLSPSPSSSGKVSAMASPPVPSSAAPRRRPGTRLSVATGGEQLVTAQEASQEPAYGVVSIHHVGILCENLERSMAFYKDLLGLKVNPARPTDKLPYRGAWLWVGSEMIHLMELPNPDPLTGRPEHGGRDRHTCMAIKDVLKLKEIFDKAGIKYTLSKSGRPAIFARDPDGNALEFTQV

>OsglyI10

MAGCRRPTTEMGEVCKRVAPSVREEEEEEENGDGGGVDPAAESSSAKLYEDVPEMPLMALNHISRLCKSIDASVRFYVKALGFVLIHRPPALDFNGAWLFNYGVGIHLVQRDDARRAPDVNPGDLDPMDNHISFQCEDMGMMEKRLNEMGIEYMKRTINEEEGSPIDQLFFKDPDGFMIEICNCENLELVPAGALGRLRLPRDRHNPPLRMAAAGNDEA

>OsglyI11

MASGSEAEKSPEVVLEWPKKDKKRLLHAVYRVGDLDRTIKCYTECFGMKLLRKRDVPEEKYTNAFLGFGPEDTNFALELTYNYGVDKYDIGAGFGHFAIATEDVYKLAEKIKSSCCCKITREPGPVKGGSTVIAFAQDPDGYMFELIQRGPTPEPLCQVMLRVGDLDRSIKFYEKALGMKLLRKKDVPDYKYTIAMLGYADEDKTTVIELTYNYGVTEYTKGNAYAQVAIGTEDVYKSAEAVELVTKELGGKILRQPGPLPGLNTKIASFLDPDGWKVVLVDNADFLKELQ

>ATGLYI-1

MAANMMRPAFAYTVVYVKDVAKSVEFYSRAFGHNVRRLDESHRWGELESGQTTIAFTPLHQHETDDLTGKVQATQSARERAPIEVCFCYPDVDAAFKRAVENGAEAVSKPEDKEWGQKVGYVRDIDGIVVRIGSHVK

>ATGLYI-2

MSSYSIASAISRISPLIRFVKPYSTGFSFITCACNSTRRPKRFDQLCVFSMASEARESPANNPGLSTNRDEATKGYIMQQTMFRIKDPKASLDFYSRVLGMSLLKRLDFSEMKFSLYFLGYEDTTTAPTDPTERTVWTFGQPATIELTHNWGTESDPEFKGYHNGNSEPRGFGHIGVTVDDVHKACERFEELGVEFAKKPNDGKMKNIAFIKDPDGYWIEIFDLKTIGTTTVNAA

>ATGLYI-3

MNEIASASMLRLCQCFISICNVHFVSMRAAESSFLLSRNMAEASDLLEWPKKDNRRFLHVVYRVGDLDRTIEFYTEVFGMKLLRKRDIPEEKYSNAFLGFGPETSNFVVELTYNYGVSSYDIGTGFGHFAISTQDVSKLVENVRAKGGNVTREPGPVKGGGSVIAFVKDPDGYTFELIQRGPTPEPFCQVMLRVGDLDRAIKFYEKALGMRLLRKIERPEYKYTIGMMGYAEEYESIVLELTYNYDVTEYTKGNAYAQIAIGTDDVYKSGEVIKIVNQELGGKITREAGPLPGLGTKIVSFLDPDGWKTVLVDNKDFLKELE

>ATGLYI-4

MKEDAGNPLHLTSLNHVSVLCRSVDESMNFYQKVLGFIPIRRPESLNFEGAWLFGHGIGIHLLCAPEPEKLPKKTAINPKDNHISFQCESMGVVEKKLEEMGIDYVRALVEEGGIQVDQLFFHDPDGFMIEICNCDSLPVVPLVGEMARSCSRVKLHQMVQPQPQTQIHQVVYP

>ATGLYI-5

MATASFRWILQLHRDVPKAARFYEKGLDFSVNVVTLRWAELQSGPLKLALMQAPSEHVMSEKGYSSLLSFTVADINTTISKLMELGAELDGSIKYEVHGKVASVRCLDGHVLGLYEPS

>ATGLYI-6

MVRIIPMAASSIRPSLACFSDSPRFPISLLSRNLSRTLHVPQSQLFGLTSHKLLRRSVNCLGVAESGKAAQATTQDDLLTWVKNDKRRMLHVVYRVGDMDRTIKFYTECLGMKLLRKRDIPEEKYTNAFLGYGPEDSHFVIELTYNYGVDKYDIGAGFGHFGIAVDDVAKTVELVKAKGGKVSREPGPVKGGKTVIAFIEDPDGYKFELLERGPTPEPLCQVMLRVGDLDRAIKFYEKAFGMELLRTRDNPEYKYTIAMMGYGPEDKFPVLELTYNYGVTEYDKGNAYAQIAIGTDDVYKTAEAIKLFGGKITREPGPLPGISTKITACLDPDGWKSVFVDNIDFLKELE

>ATGLYI-7

MKDETGNPLHIKSLNHISLLCRSVEESISFYQNVLGFLPIRRPDSFDFDGAWLFGHGIGIHLLQSPEPEKLLKKTEINPKDNHISFQCESMEAVEKKLKEMEIEYVRAVVEEGGIQVDQLFFHDPDAFMIEICNCDSLPVIPLAGEMARSCSRLNIRQLVQPTQIHP

>ATGLYI-8

MEEKKKKGDDELNSKPPLMALNHVSRLCKDVKKSLEFYTKVLGFVEIERPASFDFDGAWLFNYGVGIHLVQAKDQDKLPSDTDHLDPMDNHISFQCEDMEALEKRLKEVKVKYIKRTVGDEKDAAIDQLFFNDPDGFMVEICNCENLELVPCHSADAIRLPEDRHAPPVALPDSSNRRMPQPNS

>ATGLYI-9

MASLGHIARESSDITRLAQFYKEVFGFEEIESPDFGDLQVVWLNLPGAFAMHIIQRNPSTNLPEGPYSATSAVKDPSHLPMGHHICFSVPNFDSFLHSLKEKGIETFQKSLPDGKVKQVFFFDPDGNGLEVASRS

>ATGLYI-10

MATASFRWILQLHRDVPKAARFYAQGLDFSVNVVTLRWAELHSGPIKLALMQSPSNHVAEKGYSSLLSFTVTDINTTVTKLMALGAELDGTIKYEIHGKVAAMKCPDGYMLGLYEAA

>ATGLYI-11

MASIFRPSSASLDLRPKVICTNLSTKERFEFQKKSVRKERINVRFYSLKAKAQGSSIEGISVVQEKELNNKTDYGVVGVHHVGLLCENLERSLEFYQNILGLEINEARPHDKLPYRGAWLWVGSEMIHLMELPNPDPLTGRPEHGGRDRHACIAIRDVSNLKEILDKAGIAYTMSKSGRPAIFTRDPDANALEFTQV

>GmGLYI-1

MSSSIRPSLSSFMLPSLASCNPSQKLSLFRLGSGIRQFHKFGLKASRFLRHDDKCMRVMAFGNMSTAATQENVLDWVKHDKRRMLHVVYRVGDLDKSIKFYRECLGMKLLRKRDMQEQKYTNAFLGYGPEDAHFVVELTYSNSYGIEKYDIGDGFGHFGIAIDDISRIVELVRAKGGKITREPSPVKGGNTTIAYIEDPDGYQFELLERVPSPEPLCKVMLRVGDLDRSIKFYEKAFGMELLRTQDDPESKSTIGILGYGPEEKNTVLELTYNYGVTNYDKGDAYAQITIDTDDVYKTAEAIKLAGGKITREPGPIPVMKTKITSCVDPDGWKTVFVDNVDFRRELE

>GmGLYI-2

MKMEIEEVGNCEALPLLSLNHVSLLCRSVWVSMRFYEDVLGFVPIKRPSSFKFTGAWFYNYGIGIHLIENPNIDEFDTCVNEERPINPKDNHISFQCTDVELVKKRLEERGMRYVTAVVEEGGIQVDQVFFHDPDGYMIELCNCENIPIIPISSCSFKPRGHSFKKAAPNKCGFMENVMMESLSTDMINFSF

>GmGLYI-3

MSSSLMLPAASMLRPCTTSSSSCTSSRRLALFHLVSTGSIALPQAQLFGAKGPELLRVVEASAAEKLAQPEKDLFDWVKNDNRRFLHVVYRVGDLEKTIKFYTECLGMKLLRQRDIPEDRYSNAFLGYGPEDSNFTVELTYNYGVDNYDIGSGFGHFGVAVEDIYKRVDLVKAKGGKVTREPGPVKDGSAVIAFIEDPDGYKFELLERRPTSEPLCQVMLRVGDLDRAIAFYEKAVGMKLLRKRDNPEQKYTVAFMGYGPEDKNTVLELTYNYGVTNYDKGNGYAQIAIGTNDVYKTAEAIKLCGGKIIREPGPLPGINTKIVACLDPDGWKLAFVDNVDFLKELE

>GmGLYI-4

MVLVRVVPMASSSSIRPTLSSLRFLTPSSLSLSNPSSRISFSHLPSPSVSQSNSFGLKASRELRQHGNSTRIMASGDVSQSISAASPENVLEWVKQDKRRMLHVVYRVGDLDRTIKFYTECLGMKLLRKRDIPEEKYTNAFLGYGPEDSHFVIELTYNYGVDKYDIGTGFGHFGIAVDDVAKAVELIRAKGGKITREPGPVKGGRSVIAFIEDPDGYKFELIERGPTPEPLCQVMLRVGDLNRSIEFYEKAFGMELLRTRDNPEYKYTIAMLGYGPEDKSTVLELTYNYGVTEYDKGNAYAQIAVGTDDVYKTAEAIKLAGGKITREPGALPGINTKITACLDPDGWKSYITRSGRSVFCLLWHDT

>GmGLYI-5

LPQAQLFGAEKIAQPEKNLFDWVKNDNRRFLHVVYRVGDLEKTIKYALLRKRDIPEDRYSNAFLGYGPEESNFTVELTYNYGVDNYDIGSGFGHFGVAGRLITREPGPVKDGSAVIALIEDPDGYKFELLERRPTSEPLCQVMLRVGDIDRAAGMKLLRKRDNPEQKYTVAFMGYGPEYMNSVLELTYNYGVTNYDKGNGYAQIAIGTNDVYKTAEAIKLCGRKIIREPGPLPGINTKIVACLDPDGWKLAFVDNVDFLKELE

>GmGLYI-6

MKESVGNPLRLQSVNHISLICRSVEQSMDFYQNVLGFYPIRRPGSLDFDGAWLFGYGIGIHLLEAENPEKLPKKKEINPKDNHISFQCESMVAVEKKLKEMEIDYVRATVEEGGIQVDQLFFHDPDGFMIEICNCDSLPVIPLVGEVARSCSLVNLEKMQNQQQIQKMLQQL

>GmGLYI-7

MADLLEWSKQDKKRMLHVVYRVGDLDRTIKFYTECLGMKLLRQRDIPEEKYANAFLGFGPEESHFVVELTYNYGVTSYDIGDGFGHFAIATQDIYKLVEHIRAKGGNITREPGPVQGGTTVIAFVKDPDGYTFGLIQRPTVHDPFCQVMLRVGDLERSIKFYEKALGMKVVRKVDKPEYKYTIAMLGYGEEHETTVLELTYNYGVTEYSKGNAYAQIAIGTDDVYKSAEVVNQVIKEVGGKITRQPGPIPGLNTKTTSFLDPDGWKTVLVDNVDFLEELK

>GmGLYI-8

MVLVRLVPMASSSIRPALSTPSSFSLFSPSRRISFSHLPSPSVSQSNSFGLKASRVLRQYGNSTRIMASGDLSHSVAAASPENVLEWVKQDKRRMLHVVYRVGDLDRTIKFYTECLGMKLLRKRDIPEEKYTNAFLGYGPEDSHFVIELTYNYGVDKYDIGTGFGHFGIAVDDVAKAVELIRAKGGKITREPGPVKGGRSVIAFIEDPDGYKFELIERGPTPEPLCQVMLRVGDLNRSIEFYEKAFGMELLRTRDNPEYKYTIAMLGYGPEDKSTVLELTYNYGVTEYDKGNAYAQIAIGTDDVYKTAEAIKLAGGKITREPGPLPGINTKITACLDPDGWKSVFVDNVDFLKELE

>GmGLYI-9

MKESTMGNPLRLQSVNHISLICRSVEQSMDFYQNVLGFYPIRRPGSLDFDGAWLFGYGIGIHLLEAENPENLPKKKEINPKDNHISFQCESMEPVEKKLKEMEIDYVRATVEEGRIQVDQLFFHDPDDFMIEICNCDSLSR

>GmGLYI-10

MLLYKATRPQCRRLFWFVVLCLAIFTIFTEHLHSTLNMAEATQSNAELLEWPKKDKRRFLHVVYRVGDLDRTIKFYTECFGMKLLRKRDIPEEKYANAFLGFGPEQSHFVVELTYNYGVTSYDIGTGFGHFAIATPDVYKLVEDIRAKGGNITREPGPVKGGKSVIAFVKDPDGYAFELIQRSSTPEPLCQVMLRVGDLERSIKFYEKTLGLRVVKKTDRPEYKYTIAMLGYAEEHETTVLELTYNYGVTEYTKGNAYAQVAIGTDDVYKSAEVVNIVTQELGGKITRQPGPVPGLNTKITSFLDPDGWKTVLVDNQDFLKELE

>GmGLYI-11

MASSIRPSLSSFMLPSLRSCNPSEKLSLFHLGSGIRLYHKFGLKSSRLLRHDDNKCMRVMASGNMSTAATQENVLDWVKHDKRRMLHVVYRVGDLDKSIKFYRECLGMKLLRKRDMQEQRYTNAFLGYGPEDAHFVAELTYNYGIDKYDIGDGFGHFGLAVDDISRIVELVRAKGGKITREPSPVKGGNSTIAYIEDPDGYQFELSERVSSPEPLSKVMLRVGDLDRSIKFYEKAFGMELLRTQDDPESKSTIAILGYGPEEKNTVLELTYNYGVTDYDKGDAYAQITIGTDDVYKTAEAIKLAGGKITREPGPVPGIKTKITLCVDPDGWKTVFVDNVDFRRELE

>GmGLYI-12

KEGKGKEENPPPLLAMNHVSRLCRNVKESIDFYTKVLGFVLIERPQALDFEGAWLFNYGVGIHLCEDLEAMEKKLKEKNVKYMKRTLEREDGTTMDQIFFNDPDGFMVEI

>GmGLYI-13

MKMEIEEVGNCEALPLLSLNHVSLLCRSVWESMRFYEDVLGFVPIKRPSSFKFTGAWFYNYGIGIHLIENPNIDEFDTCVVEERPINPKDNHISFQCTDVELVKKRLEERGMRYVTAVVEEGGIQVDQVFFHDPDGYMIELCNCENIPIIPISSCSFKPRGHSFKKAAPNKCGFMENVMMESLSTDMINFSF

>GmGLYI-14

MKDSSSAMVHSMRRSFFNFCLTEKAQLDLYGHSNRINCDCSEPKESPSNNPGLHTTPDQATKAYFTQQTMFRIKDPKVSLDFYSRVLGTYLLKRLDFLEMKFSLYFMGYEDTTKAPSNPVERTVWTFSQKATMELTDNWGTENDPEFKGYHNGNSEPLGYGHIGIAVDDTYKACERFQNLGVEFVTKPDDGFFSQHYCFKHYFQVFFILELFSAGEIKGLAFIKDPDGYWIELFDLKILGGEQAAAHA

>GmGLYI-15

MTVTASLHRLSRLRFIAKPQPFLSPHSIPSHFSLTPKTKKANRFRFLSMAAEPKESPSNNPGLHTTPDEATKGYIMQQTMFRIKDPKVSLDFYSRVLGMSLLKRLDFPEMKFSLYFMGYENTAEAPSNPIDKVVWTFSQKATIELTHNWGTESDPEFKGYHNGNSEPRGFGHIGVTVDDTYKACERFQNLGVEFVKKPEDGKMKGIAFIKDPDGYWIEIFDRKTIGNVTQTAA

>GmGLYI-16

MAATASLHRLSRLRFIAKPQPFLSPHSTPSHFSLTPKTKKPNRFRFRFRSMAAEPKESPSNNPGLHTTPDEATKGYIMQQTMFRIKDPKVSLDFYSRVLGMSLLKRLDFPEMKFSLYFMGYEDTTEAPSNPIDKVVWTFSQKATIELTHNWGTESDPEFKGYHNGNSEPRGFGHIGITVDDTYKACERFQNLGVEFVKKPDDGKMKGIAFIKDPDGYWIEIFDRKTIGNVTQAPA

>GmGLYI-17

MEKMELAETPLPLLSLNHVSFVCKSVSESVKFYEDVLGFLLIKRPSSFKFEGAWLFNYGIGIHLLESEKVPVKKREINPKENHISFQCSDMKVIMQKLDAMKIEYVTAVVEEGGIKVDQLFFHDPDGYMIEICNCQNLPVLPISSCPLKQLGGEATFKINCFAEESMSMLMMDNFVMDMLKISI

>GmGLYI-18

MSCCSCSAMAFLLKAPSFLPPLNQKLNYTHKSFSPINLQSKFYHASVRNGRWNVPSMTIKAQAAVEGDVLLDEESICVNEESDYGVVCMHHVGILCENLERSLDFYQNVLGLKINEARPHNKLPYRGAWLWVGSEMIHLMELPNPDPLTGRPQHGGRDRHTCIAIRDVSKLKAIFDKAGIAYTLSHSGRPAIFTRDPDANALEFTQVDD

>GmGLYI-19

MANPLQLKSLNHISIVCASVEKSVDFYVNVLGFSPIKRPSSLDFNGAWLFNYGIGIHLLQSENPEGMPKTAPINPKDNHISFQCESIAAVEKRLQQMKIEYVKNRVEESGTYVDQLFFHDPDGMMIEICNCDNIPVVPLTEDKVWSCSRFNCNIQNHQQQIQQMIPM

>GmGLYI-20

MKENVGNPLHLKSVNHISLICTSVKESINFYQNLLGFFPIRRPGSFDFDGAWLFGYGIGIHLLQAEDPDNVPRKTKINPKDNHISFQCESMGAVEKKLGEMEIEYVHATVEEGGIKVDQLFFHDPDGFMIEICNCDSLPVIPLAASGNNNGMVRSCSRLNLQILQQIHQFLNQ

>GmGLYI-21

MAEATQSNAELLEWPKKDKRRFLHVVYRVGDLDRTIKFYTECFGMKLLRKRDIPEEKYANAFLGFGPEQSHFVVELTYNYGVTSYDIGTGFGHFAIATPDVYKLVEDIRAKGGNVTREPGPVKGGKSVIAFVKDPDGYAFELIQRPSTPEPLCQVMLRVGDLERSIKFYEKALGLRVVKKTDRPEYKYTIAMLGYAEEHETTVLELTYNYGVTEYTKGNAYAQVAIGTDDVYKSAEVVNIVTQELGGKITRQPGPIPGLNTKITAFLDPDGWKTVLVDNQDFLKELE

>GmGLYI-22

MGKMEPLPLLSLNHVSFVCKSVSESVKFYQDVLGFVLIKRPSSFKFEGAWLFNYGIGIHLLESEKVPVEKREINPKENHISFQCSDMKVIMQKLDAMKIEYVRAVVEEGGIKVDQLFFHDPDGYMIEICNCQNLPVLPISSCPLKQLAAGEATTLNINCFADESVSMLMMDNLVMDMLKISI

>GmGLYI-23

MNCCSAMASLLKSPSFLSPLNQKLNYVSFSPMTTNLQSKFCRASVRNGRWHVPSLTIKSQAAVEGDVLEKESVSINEESDYGVVCMHHVGILCENLERSLEFYQNVLGLKINEARPHDKLPYRGAWLWVGSEMIHLMELPNPDPLTGRAQHGGRDRHTCIAIRDVSKLKAIFDKAGIPYTLSHSGRPAIFARDPDANALEFTQVDG

>GmGLYI-24

MANPLQLKSLNHISIVCASVEKSVDFYVNVLGFSPIKRPSSLDFNGAWLFNYGIGIHLLQSEDPEGMPKLVPINPKDNHISFQHSSGGKRLQQMKIEYVKNRVEENGMMIEICNCDNIPVVPLPEDKVWSCSRFNCNIQNRQQQI

>Medtr1g022325.1(MtGLYI-1)

MGNETQTKTGFKLVGFKKFIRTNPKTDRFKVKRFHHVEFWCTDATNTALRFSQGLGMPIVAKSDLSTGNLIHASYLLRSGDLNFLFSAPYSPSISLSSPSSTASIPTFSASTCFSFCASHGLAVRAIAIEVDDAELAFTVSVNHGALPSSPPIVLENGVKLAEVHLFGVDVVLRYVSYNNPNLLFLPGFESLLNESSNSSLDFGIRRLDHANANVPELASAVKYIKQFTGFHEFAEFTTEDVGTSESGLNNVVLASNDETVLLPICEPIYGTKRKSPIETYLEHNEGAGFQHLALASEDIFRTLREMRKKSGVGGFEFMAPPPVTYYRNLKNRVVDVLSDEQIKECEELGILVDRDDQGTILQIFTKPVGDRPTVLIEIIQRVGCMLKDEEEKEYQRGGCGGFGKGNFSELFKSIEEYEKTLETRRTA

>Medtr1g115170.1(MtGLYI-2)

MKETVAFYEKVLEFISIVRPGSFDFGGAWLFGHGIGIHLLLAEDPEKIPRKNEINTKDKHISFQCDESMDAVEKYLKDMKIGLKRAMVEENGIQVDQLFFHDPDGFMIEICNCDSLPVIPLAGGMVTLCPRLNFESMPQQIDQVAKQI

>Medtr2g005880.1(MtGLYI-3)

MPLPLLSLNHVSFVCRSLQESVKFYENVLGFVLIKRPSSFKFQGAWLFNYGIGIHLLETESDKVPVKRGEINTKENHISFQCSDMKLIMKNLDEMNIEYKTAVVEDGGIKVDQLFFHDPDGYMIEMCNCQNLPVLPISTCPLKQPTNQAPVPFYGEGKNCHAEEALLMMEILVIDLLRISI

>Medtr2g023500.1(MtGLYI-4)

MAEAAQPNAELLEWAKKDKRRFLHAVYRVGDLDRTIKFYTEAFGMKLLRKRDVPEEKYANAFLGFGPETSNFVVELTYNYGVTSYDIGTGFGHFAIATPDVYKFVENARAKGGKVTREPGPVSGGTSVIAFVADPDGYLFEILQRASTPEPLCQVMLRVGDLERSIKFYEKALGLKLARTIDRPQYKYTLAMLGYAEEHETIVLELTYNYGVTEYTKGNAYAQVAVGTDDVYKSAELVNLATQEFGGKITRQPGPIPGLNTKITSFLDPDGWKTVLVDNQDFLKELE

>Medtr2g103460.1(MtGLYI-5)

MDAIVGNPLRLKSVNHISLICRSVDVTVAFYENVLGFVSIVRPGSFNFEGAWLFGHGIGIHLLKAEDPEKIPRKKEINTKDNHISFQCDGSIDAVEKYLNDKKIVCKRALVEENGIQVDQLFFHDPDGFMIEICNCDSLPVIPLAGEIVNSCSRINLETMPQKIHQPVEKI

>Medtr2g103490.1(MtGLYI-6)

MKEIVGNPLRLKSVNHISLICKSVNESVSFYEKVLGFISIVRPGSFDFEGAWLFGYGIGIHLLQAEDPENIPRKNEINPKDNHISFQCDESMDTVEKYLNDKKIGCKRAMVEENGIQVDQLFFHDPDGFMIEICNCDSLPVIPLAGEMVRSCSRLNLEIMPQQIHQVVKQI

>Medtr3g110185.1(MtGLYI-7)

MHAIAMDVYGKRSLLTLMDKEKTEQQESPNIHLHVQIHSHREANEQDIQFSPPRPSTTFPQSPWTLSSLPPPSPSLLYHCIASLHRHEGNIYSIAVSKGFIFTGSNSSRIRVWKQPDCMDKGYLKSNSGEIRTILAYNNMVFSSHKDHKIRIWNFNVSENFKSKKVATLPKRSKNSFLNFSRTKNNNSHNHKHKDLVSCMAYYHSEGLLYTGSHDRTVKAWRISDRNCVDSFLAHEDHVNAILVNQDDGCVFTCSSDGSVKIWRRVYTENSHTLTMTLKFQHSPVNTLALSSSFNHCFLYSGSSDGMINFWEKERLCYRFNHGGFLQGHRFAVLCVETVGNMVFSGSEDTTIRVWRREEDSCYHECLMVLDGHRGPVRCLAACLEMEKVVVGFLVYSASLDQTFKVWRIKVFSEDENVCLDGDNNKCDGRVKKIREYDMSPVLSPSWVEKKLQGGNKETDKGRSVSFICITMISSLMLPSATTLRPCCSCSITPSSSSSSSRRIALFHLLTTGGIALPQSQLLGGKGSDLFQIAEANAAVNLAQPDQNLFNWVQNDNRRFLHVVYKVGDLDKTIKFYTECLGMKLLRKRDIPEDKYSNAFLGYGPEDSSFTVELTYNYGVDNYDIGTGFGHFGIIAEDVSKTVDIVKAKGGKVTREPGSVKGGSIVTASVEDPSGYRFELLERRPTREPLCKVMLRVGDLDRVIAFYEKAVGMKLLHKIDNPEEKYTVAKLGYGPEANGPVLQLTYNYGVTNYDKGNGYAQIAIGTDDVYKTAEAIKSCGGKIIREPGPLPGINTKIVVCLDPDGWKLVFVDNVDFLKELE

>Medtr4g011020.1(MtGLYI-8)

MKESVGNPLHLKSVNHISLICRSVEESIDFYQNVLGFFPIRRPGSFDFDGAWLFGYGIGIHLLEAENPETLPRKKEINPKDNHISFQCESMGAVEKKLKEMEINYVRARVEEGGIEVDQLFFHDPDGFMIEICNCDSLPVIPLVGEVARSCSRLNLHIMQNQNQNQQNQIHKIVK

>Medtr4g039890.1(MtGLYI-9)

MCVKFDRFYTECLGMKLLRKRDIPEDKYSNAFLGYGPEDSSFTVELTYNYGMDNYDIGTGFGHFGIIAEDVSKTVDIVKAKGGKVTREPGSVIGGSIVTASVEDPSGYRFKLLERRTTREPLCKVMLRVGDLDRVIAFYEKAVGMKLLHKIDNPEEKYTVAKLGYGPVLELTYNYGVTNYDKGNGYAQIAIGTDDVYKTAEAIKSCGGKVIREPGPLPGINTKIVVCLDPDGWKLVWHFSIFSLYLM

>Medtr4g057685.1(MtGLYI-10.1)

MMSIATSNFLSRFRFIAKHQSLPIRSPVSIPFHFSLKKQPIRRFRFFSMAASESKESPANNPGLHATVDEATKGYFMQQTMFRIKDPKVSLDFYSRVLGMSLLKRLDFPEMKFSLYFMGYEDTSEAPSNSVDRTVWTFAQKATIELTHNWGTESDPEFKGYHNGNSDPRGFGHIGITVDDTYKACERFQNLGVEFVKKPEDGKMKGIAFIKDPDGYWIEIFDRKTIGNVTGSAA

>Medtr4g063500.1(MtGLYI-11.1)

MASKLSPEFAYTVLYVKDVAESVAFYSKAFGYSVRRLDESHRWGELESGHTTIAFTPIHQHETDDLTGVVHTTRSNKERPPVEVCFVYTDVDAAYKRAVENGAVPVSEPEMKEWGQKVGYVRDIDGIVIRMGNHVKPAKLD

>Medtr4g114080.1(MtGLYI-12)

MGNPLQLKSLNHISLVCRSLDKSVDFYVNVLGFFPIKRPTSLAFNGAWLFNYGIGIHLLQSDDPESMTKNVHINPKDNHISFQCESMAAVENKLQQMKIEYVKNLVEENGIYVDQLFFHDPDGTMIEICNCDNIPIVPLSENSTIWSCSRFNCNIQNQQQQIQQMISM

>Medtr4g125860.1(MtGLYI-13.1)

MASLLKVSSFISPLHHKLNYVSFSPKFNHVSVRNERWNAPSITVKAQTAVEGDVINNESLSSNEQSDYGVVSVHHVGILCENLERSLDFYQNVLGLKINEARPHDKLPYRGTWLWVGSEMIHLMELPNPDPLTGRPQHGGRDRHTCIAIRDVSKLKAILDKAGVPYTLSRSGRPAIFTRDPDANALEFTQIDD

>Medtr4g132260.1(MtGLYI-14.1)

MAEIDLEWPKKDNRRLLHVVYRVGDLERTIKFYTEALGMKLLRQRDVPEEKYANAFVGFGDEHSHFAVELTYNYGVTSYDVGDGFGHFAIATQDVYKLVEHIRAKGGNITREAGPVQGGTTVIAFVKDPDGYTFALVQRPIVHDPFCQISLRVGDLERAIKFYEKALGLKVVRKVDNPENKYTIAILGYKEEDDATVLELTYNYGVTEYSKGTAYAQIAIGTDDVYKSADVVNLVTQELGGEITLQPGPIPGLNTKVTSFLDPDGWKTALVDNEDFLKELE

>Medtr4g132270.1(MtGLYI-15.1)

MSGFCVGLSLPTDIPHSLSHLVVMAEIDLEWPKKDNRRLLHVVYRVGDLERTIKFYTEALGMKLLRQRDVPEEKYANAFLGFGDEQSHFVVELTYNYGVTSYDVGDGFGHFAIATQDVYKLVEHIRAKGGNITREAGPVQGGTTVIAFVKDPDGYTFALVQRPIVHDPFCQISLRVGDLERAIKFYEKALGLKVVRKVDNPENKYTIAILGYKEEDDATVLELTYNYGVTEYSKGTAYAQIAVGTDDVYKSADVVNLVTQELGGKITRQPGPIPGLNTKVVSFLDPDGWKTVLVDNEDFLKELE

>Medtr5g006360.1(MtGLYI-16.1)

MAGVCLNHISRESNDINRLAKFYQEIFGFEEVESPKFGEFKVVWLRVPSSSLYLHLIERNPSNNLPEGPWSATSPVKDPSHLPRGHHLCFSVSNFQSFLQTLKDKGIETFEKSLPNGKIKQVFFFDPDGNGLEVASKEDS

>Medtr5g006370.1(MtGLYI-17.1)

MAAAEGVSLNHIARESTDVKRLSKFYQEMFGFEEVETPDFGELKIIWLRLPSSSLLIHLIQHSNGELAPSSSIPVKDPSHIRLGHHLCFSISNLHSFHNTLKDKGIETFETTNGNIKRVFFYDPDGNELEVFASIEDSS

>Medtr5g009740.1(MtGLYI-18.1)

MAASFRWLLQLHKDVPKAARFYSEGLDFTVNVCTLRWAELQSGPLKLALMHSPIDQSTQKGYSSLLSFTVTDINSTVTKLMALGAELDGPIKYEVHGKVAAMRCIDGHLLGLYEPV

>Medtr5g021610.1(MtGLYI-19.1)

MQLLDSLREQELKMEIEEVCEAQALPLLSLNHVSLLCRSVLESMQFYEDVLGFVPIKRPSSFKFTGAWFYNYGIGIHLIQNPDIDEFDTYMNESRPINPKDNHISFQCTDVELVKKRLEEKGMRYVTALVEDEGIKVDQVFFHDPDGYMIELCNCENIPIIPISSCTASFKPRSHSFKRSTSNFKCGGFMQNVMMQSLSMDMMNFAF

>Medtr5g090990.1(MtGLYI-20.1)

MANETHNQTGFKLVGCKNFIRTNPKTDRFKVKHFHHVEFWCTDATNTAHRFSHGLGMPIVAKSDLSTGNLTHASYLLRSGDLNFLFTAAYSPSISLSSPSSTASIPTFSPSTCFSFSNSHGLNVRALAVEVEDAELAYTVSVSYGALPSSPPVVLENGVKLAEVRLFGDVVLRYVSYNNPNQNQNLLFLPGFETLSGESSNSSLDFGIRQLDHANGNVPELSSALKYIKQFTGFHDFAEFTAEDVESGLNAVALANNDETVLLPLCEPVYGTKRKSTIETYLEHNEGAGFQHLALASEDIFKTLREMRKRSGVGGFEFMPSPPVTYYRNLKNRVGDVLSDEQIKECEELGILVDRDDQGTLLQIFTKPIGDRPTIFLEIIQRVGCMLKDEEGKEYQKGGCGGFGKGNFSELFKSIEEYEKTLETRRTA

>Medtr5g091060.1(MtGLYI-21.1)

MAIETETQTQTQTGFKLVGFKNFVRANPKSDRFNVKRFHHVEFWCTDATNTARRFSHGLGMPIVAKSDLSTGNLTHASYLLRSGDLNFLFSAAYSPSISLSSPSSTAAIPTFSASTCFSFSASHGLAVRAVAVEVEDAEVAFTTSVNLGAIPSSPPVILENNVKLAEVHLYGDVVLRYVSYNDLNPNQNPNLFFLPGFERVSDESSNSSLDFGIRRLDHAVGNVPELSSAVKYVKQFTGFHEFAEFTAEDVGTSESGLNSVVLANNEETVLLPMNEPVYGTKRKSQIETYLEHNEGAGLQHLALMSADIFRTLREMRKRSGVGGFEFMPSPPVTYYRNLKNRVGDVLSDEQIKECEELGILVDRDDQGTLLQIFTKPIGDRPTIFIEIIQRVGCMLKDEEGKEYQKGGCGGFGKGNFSELFKSIEEYEKTLETRRTA

>Medtr6g087120.1(MtGLYI-22.1)

MASSSIRPSLSSLNKLPSFSSRNLSQRFSLFHLRNGVRLLPQNFGLKASRLLRHDSGSMRVMASRSMSQSVTQENALDWVKWDKRRMLHVVYRVGDFDKSIKFYTECLGMKVLRKRDMTEEKYTNAFLGYGPEDAHFAIELTYNYGIETYDIGTGFGHYGIAMDDISRVVDIVRAKGGIITREPGPVKGGDSTVAVIEDPDGYKFELLERAPSPEPLCKVMLRVGDLDRSIKFYEKVVGMELLRKQDDPESKCTVAIMGYGPEEKTTVLELTYNYGITKYDKGDAYAQIAIGTDDVYKTAEAIKLAGGKITREAGPVPGYRTKITSCVDPDGWKTVFVDNHDFHKELE

>Medtr8g076160.1(MtGLYI-23.1)

MGIQEIGSYEAPLPLLSLNHVSILCRSVLDSMRFYEEILGFGLIKRPSSFKFNGAWLYNYGFGIHLLENPNYDEFDTPMSESRPINPKDNHISFQCTDVGLVKMRLEDMGMKYVTALVEDEGIKVEQVFFHDPDGYMIELCNCENIPIVPISSASGSFKARGQSFKKTVSNKCGFMENVMMRSLTKDMMNFAF

>Medtr8g102980.1(MtGLYI-24.1)

MVRVIPIASSSILPTLSLFNRTPRISFSHFSTAVPQSHNFGLKACRLFKQNGNSLKVMSSGNVSSSVTAASPENVLEWVKQDKRRMLHVVYRVGDLDRTIKFYTECLGMKLLRKRDIPEERYTNAFLGYGPEDSHFVIELTYNYGVDKYDIGTAFGHFGIAVDDITKTVELIRAKGGKITREPGPVKGGKTVIAFVEDPDGYKFELLERGPTPEPLCQVMLRVGDLNRSIEFYEKAFGMELLRTRDNPDNKYTIAMLGYGPEDKSTVLELTYNYGVTEYDKGNAYAQIAIGTDDVYKTAEAIKLSTGKLTREPGPLPGINTKITACLDPDGWKTVFVDNIDFLKELE

>Medtr0003s0630.1(MtGLYI-25.1)

MQKQEVKEEERNSNKKEEKNEKEGDEGTKESNQTPLMALNHISRLCRDVKESIDFYTKVLGFVLIERPQVLDFEGAWLFNYGVGIHLVQSKEEQKLPSPDAQHDLDPQDNHISFQCEDVKGMEKKLKEMKVKYKKRNLEAEDGTTMDQIFFNDPDGFMVEICNCENLKLTPADSQGKIKIPMDRHTPPVETNQNEHDNVK

>Medtr0430s0010.1(MtGLYI-26.1)

VAKTVNIVKEKWGKVMRELEPVKDGSTVTAFIEDPSGDRFELLGRRLTREPLCKVMLQADNLDCVIAFYEKAVGMKLLHKIVNPK

>Medtr1239s0010.1(MtGLYI-27.1)

MGTIRALGYVGFTATNIDRWKDFAPGVLGLQLSETWPDGTLVLRADAYQRRIFIHPGRVDEIRYIGWEVYDADSLEKLKSQLTGKRVPFVDLNEDETAHRAVIDGLKFLDTDGLHIEAFYGASQSKHEPFVSPVGQGPFVTGEQGLGHIVVHPENYSAAVAFYKDVLDFKISDYCTINTLGARDGHATFMHVNPRHHSLALANFPIGQRLNHIMLELETIDDVGCAYERALAAGAHILLDLGRHTNDDVFSFYVMTPSGWSVEIGCGGRRIDDTTWHVSHHTRPSSWGHNLDSTSRMVKVGNLDIHYHDSGSGDQVVVLLHGGGPGASSWSNFQRNIGPLSEHFRVLAIDMPHFGKSTKPEGRYLDRPWYAEVVGATLDTLNISKAHFVGNSLGGSVSMVLSVERPEIVDRLVLMGTMGSLPVFAPLPPEGAKHIIEYYHGEGPTREKLEAFLRSMIYDQNLITTDFIEERFIASTTPELLFVAQQPKLNTHFTQWQTADQVKHKSLLLYGRDDRVVPWDTSLLLLRLMPNADLHIFSRCGHWAQWERADEFNSVVANFL

>Medtr1275s0010.1(MtGLYI-28.1)

MLLGSRTDIRIAGESAEFGFTEIRHGLGGPAAIISRLRDQIPYTSLMWLAFGQHINAHEAHRIGLVNELVPDDQVLDRAMEVARAIAEVPPLAIRAEKQSLLRTQHQPFKEAVQYGTALFSMIQMSADAREGVQAFVEKRRPNFRMDRRREPAGRWRAGRFNAGSFMSRVVVVTGAASGNGLAIASRFLDHGDRVVAVDVSTDGLGARFQKEWRPYEERVIALTKDVSCQTDIDASRASQDLSFRVRPSTRGEGARGIPCMPEQCEGVVVNIASIATLVANPGRTSYATSKGALLQRTRSIAADYAHAGIRCNALCPGLIETAMTQWRRGGSEQRNQVLAKIPQNEVGTVDDVASAVMFISDPQSRYFNGAALVIKADHAAYVVSSLKEALTFWVDGMGATLEGRFKAGGPMLANVTGAVGADVSIALIEIAGQRLELLEYQGVTPNPGATLRPYDAGAMHLALNVDDVHAALRHVAQYGYRAQGVPQKAPTGSTAMYVVGPDGATIEFRQPEVA

>Medtr1759s0010.1(MtGLYI-29.1)

MKKIATVLFSALMLASTCASAQALCKAGKIDKIETDTTGNLLVTINDGVYSFSAKEVYSIIYQAYSENRNLFIYGNNCANGSPATRFAIPPPRAGVPILRDMAPACPRPVHPSAIPVPVPMKPAGLPLRISRRPAFGASTASSRRSRRAGCPASLRRRPSGPGHEQTPGCRISPCADRTHPSRRRPMIDHVYISVTDIEKSLAFYAEALKPLGWRIFGNYDSASGPESVPDLYGIGDDVYGKGAGVGSSIWLRKRHPGETGLYVGIVCDTNELVDAAYAAAIKAGGIDEGKPADRTYFAPGYYAANVADFDGNRLEFVHKAWNPKRHA
