# Supplementary material for: Genome-Wide Identification of Glyoxalase Genes in Medicago truncatula and Their Expression Profiling in Response to Various Developmental and Environmental Stimuli
Source: Front Plant Sci. 2017 Jun 1;8:836. doi: 10.3389/fpls.2017.00836 (PMC5452422; doi:10.3389/fpls.2017.00836)
Supplement: Supplementary file 2 [file Data_Sheet_2.DOCX]

>ATGLX2-4

MQAISKVSSAASFFRCSRKLVSQPCVRPCVRQLHVRKGLVSGVMKLFSSPLRTLRDAGKSVRISRFCSVSNVSSSLQIELVPCLTDNYAYILHDEDTGTVGVVDPSEAVPVMDALQKNSRNLTYILNTHHHYDHTGGNLELKDRYGAKVIGSAADRDRIPGIDVALKDADKWMFAGHEVHIMETPGHTRGHISFYFPGARAIFTGDTLFSLSCGKLFEGTPEQMLASLQRIIALPDDTSVYCGHEYTLSNSKFALSIEPTNEVLQSYAAYVAELRDKKLPTIPTTMKMEKACNPFLRTENTDIRRALGIPETADEAEALGIIRRAKDNFKA

>ATGLX2-3

MVMTHFSRLRQLLLLQPKFLSSQPRPLRSPPPTFLRSVMGSSSSFSSSSSKLLFRQLFENESSTFTYLLADVSHPDKPALLIDPVDKTVDRDLKLIDELGLKLIYAMNTHVHADHVTGTGLLKTKLPGVKSVISKASGSKADLFLEPGDKVSIGDIYLEVRATPGHTAGCVTYVTGEGADQPQPRMAFTGDAVLIRGCGRTDFQEGSSDQLYESVHSQIFTLPKDTLIYPAHDYKGFEVSTVGEEMQHNPRLTKDKETFKTIMSNLNLSYPKMIDVAVPANMVCGLQDVPSQAN

>ATGLX2-5

MQTISKASSATSFFRCSRKLSSQPCVRQLNIRKSLVCRVMKLVSSPLRTLRGAGKSIRVSKFCSVSNVSSLQIELVPCLKDNYAYILHDEDTGTVGVVDPSEAEPIIDSLKRSGRNLTYILNTHHHYDHTGGNLELKDRYGAKVIGSAMDKDRIPGIDMALKDGDKWMFAGHEVHVMDTPGHTKGHISLYFPGSRAIFTGDTMFSLSCGKLFEGTPKQMLASLQKITSLPDDTSIYCGHEYTLSNSKFALSLEPNNEVLQSYAAHVAELRSKKLPTIPTTVKMEKACNPFLRSSNTDIRRALRIPEAADEAEALGIIRKAKDDF

>ATGLX2-1

MPVISKASSTTTNSSIPSCSRIGGQLCVWPGLRQLCLRKSLLYGVMWLLSMPLKTLRGARKTLKITHFCSISNMPSSLKIELVPCSKDNYAYLLHDEDTGTVGVVDPSEAAPVIEALSRKNWNLTYILNTHHHDDHIGGNAELKERYGAKVIGSAVDKDRIPGIDILLKDSDKWMFAGHEVRILDTPGHTQGHISFYFPGSATIFTGDLIYSLSCGTLSEGTPEQMLSSLQKIVSLPDDTNIYCGRENTAGNLKFALSVEPKNETLQSYATRVAHLRSQGLPSIPTTVKVEKACNPFLRISSKDIRKSLSIPDSATEAEALRRIQRARDRF

>ATGLX2-2

MKIFHVPCLQDNYSYLIIDESTGDAAVVDPVDPEKVIASAEKHQAKIKFVLTTHHHWDHAGGNEKIKQLVPDIKVYGGSLDKVKGCTDAVDNGDKLTLGQDINILALHTPCHTKGHISYYVNGKEGENPAVFTGDTLFVAGCGKFFEGTAEQMYQSLCVTLAALPKPTQVYCGHEYTVKNLEFALTVEPNNGKIQQKLAWARQQRQADLPTIPSTLEEELETNPFMRVDKPEIQEKLGCKSPIDTMREVRNKKDQWRG

>OsGLYII-1

MVALLRSCRRLIPHLSACAAAAPSSSSSCAPRARPISRGLRLLPVVLAMAGYSSGSAAEGRRLLFRQLFEKESSTYTYLLADVGDPEKPAVLIDPVDRTVDRDLNLIKELGLKLVYAMNTHVHADHVTGTGLIKTKLPGVKSVIAKVSKAKADHFIEHGDKIYFGNLFLEVRSTPGHTAGCVTYVTGEGDDQPSPRMAFTGDALLIRACGRTDFQGGSSDELYESVHSQIFTLPKDTLLYPGHDYKGFTVSTVEEEVAYNARLTKDKETFKKIMDNLNLAYPKMIDVAVPANLLCGIQDPPPSKV

>OsGLYII-2

MKIIPVACLEDNYAYLIVDESTKSAAAVDPVEPEKVLAAAAEVGVRIDCVLTTHHHWDHAGGNEKMAQSVPGIKVYGGSLDNVKGCTDQVENGTKLSLGKDIEILCLHTPCHTKGHISYYVTSKEEEDPAVFTGDTLFIAGCGRFFEGTAEQMYQSLCVTLGSLPKPTQVYCGHEYTVKNLKFILTVEPDNEKVKQKLEWAQKQREANQPTIPSTIGEEFETNTFMRVDLPEIQAKFGAKSPVEALREVRKTKDNWKS

> OsGLYII-3

MRMLSKACSLVASSLPRCSSSAAPTIRGQPSLLPSVRKEWLGKPLLYGIGTLLVMPLRTLHGVGRMFGAGRFLCNMTSVSSSLQIELVPCLQDNYAYILHDVDTGTVGVVDPSEATPIINALEKRNQNLTYILNTHHHYDHTGGNLELKAKYGAKVIGSAKDRDRIPGIDITLSEGDTWMFAGHQVLVMETPGHTSGHVCYHFPGSGAIFTGDTLFSLSCGKLFEGTPQQMYSSLQKIIALPDETRVYCGHEYTLSNSKFALSIEPGNKDLQEYAANAADLRKRNTPTVPTTIGREKQCNPFLRTSSPEIKNTLSIPDHFDDARVLEVVRRAKDNF

>GmGLYII-1

RKDENPSHTNYSYLYVVVFVTNLQDNVKGCTDKVENGDKVSLGPDVTVLALLTPCHTQGHISYYVTGKEDEQPAVFTGDTLFIASCGKFFEETAEQMYQSLNVTLASLPKSTRVYRGHEYSVNNLQFAVTLEPDNLRIQKKLAWARNQWQAGQATIPSTIEDELETNPFMRVDLPEIQERVGCKSPVKALGEIRKQKDNWRG

>GmGLYII-2

MKIYHVPCLRDNYSYLIVDKSTKEGAVVDPVEPQKVLEAANSHWVNLKLVLTTHHHGDHAGGNEKIKQLVPGIKVYGSLIDNVIGCTDKVENGDKESLGADIYILCLHTPCHTKGHISYYVTGKEEEQPAVFTGDTLFIADCGKFFKGTAEQMYQSLCVTLGSLPKPTRVYCGHGEKVGCKSPVEALRELRKLKDNWKG

>GmGLYII-3

IVDESTKEGAVVDPVEPQKVLEAANSHGVNNLKLVLTTHHHGDHAGGNEKIKQLVLGMKVYGGSMDNIKGCTDKVENGDKMSLGADINILCLHTPCHTKGHISYCVTGKEEEVLRKEYNKLILKAMPKFIAGCGKFFEGTAEQIYQSLCVTLGSLPKPTRVYCGHEYAVRNLLFALTIEPDNLRIQQKLTWAKNQQQAGQSTIPSTIEEEMETNPFMRVNLPEIQGASLPVEALRELRKLKDKWKGVMELTNYCILHV

>GmGLYII-4

MGDTKERSGFCVWPDARQLCLGKGLLYGFMRLFSIPLKTLRGASRSLRVNQFCSVVNLSSSLQIELVPCLRDNYAYLLHDVDTGTVGVVDPSEAAPIIDALSKKDLNLTYIMNTNHHPDHTGGNAELKERYGAKVIGSEIDKERIPGIDIYLSDGDNWMFAGHEVHILATPGHTEGHVSFYFPGSGAIFTGDTLFSLSCGKLLEGTPKQMLSSLKRIMSLPDDTSIYCGHEYTSSNSKFALSIEPENKELQSYAAHVANLRNKGLPTIPTTVKVEKACNPFLRTWSMEIRQKLNIATTADDAEALGVIQQAKDNF

>GmGLYII-5

MNVLVFERSGFCVWPDARQLCLRKGLLYGFMRLFSIPLKTLRGASRSLRVDQFCSVVNLSSSLQIELVPCLRDNYAYLLHDVDTGTVGVVDPSEAAPIIDALSKKDLNLTYILNTNHHPDHTGGNAELKERYGAKVIGSEIDKERIPGIDIYLSDGDNWMFAGHEVHILATPGHTEGHVSFYFPGSGAIFTGDTLFSLSCGKLLEGTPEQMLSSLKRIMSLPDDTSIYCGHEYTLNNSKFALSIEPENKELQSYATHVSNLRNKGLPTIPTTLKVEKACNPFLRTWSIEIRQKLNIAATADDAEALGVIRQAKDNF

>GmGLYII-6

MPIATKLYASNVTSTLNSKTNSNNGDLIYVILIVIPVIKLFLYSTIHNQNPTTSTLQSLSSIQPFWWQLLFMLQKMLRLHFTTALSHFASKASPFPLTPVSVTVSRAIVCNNPTRFRSQMGSFSTSSSSSSKLLFRQLFEKESSTYTYLLADASHPEKPALLIDPVDRTVDRDLSIIEQLGLKLVYAMNTHVHADHVTGTGLIKSKVPSVKSVISKASGATADLYVEPGDKVQIGDLFLEVRATPGHTKGCVTYVTGDAPDQPQPRMAFTGDTLLIRGCGRTDFQGGSSEQLYKSIHSQILTLPKSTLIYPAHDYKGFTVSTVGEELQNNPRITKDEETFKNIMGNLNLSYPKMIDIAVPANMVCGIQSNPKQAEAS

>GmGLYII-7

MLHMFSKASSAMATFPCSRVKSGLCVWPDVRQLCFRKGMLYGFMRLFSTPLKTLRGASRSLRVTQFCSVANMSSSLQIELVPCLKDNYAYLLHDVDTGTVGVVDPSEAVPIIDALSRKNRNLTYILNTHHHHDHTGGNVELKARYGAKVIGSGTDKERIPGIDIHLNDGDKWMFAGHEVRVMDTPGHTRGHISFYFPGSGAIFTGDTLFSLSCGKLFEGTPQQMLSSLKKIMSLSDDTNIYCGHEYTLNNIKFALSIEPENEELQSYAAQVAYLRSKGLPTIPTTLKVEKACNPFLRTSSAAIRQSLKIAATANDAEALGVIRQAKDNF

>GmGLYII-8

MRIHHIACLQDNYSYLIVDESTKEAAAVDPVEPEKVLEVASSHGLTLKFVLTTHHHWDHAGGNDKIKQLVPGIKVYGGSIENVKGCTDKVENGDKVSLGAEITILALHTPCHTQGHISYYVTGKEDEQPAVFTGDTLFIASCGKFFEGTAEQMYQSLNVTLASLPKSTRVYCGHEYSVNNLQFALTLEPDNLRIQQKLTWARNQRQAGQATIPSTIEDELETNPFMRVDLPEIQERVGCKSPVEALGEIRKQKDNWRG

>GmGLYII-9

MLSKPSSAMPTFPSSMVRSGLCVWPNVRQLCFRKGILYGFMRLFSTPLKTLRGASRSLRVAQFCSVANMSSSLQIELVPCLKDNYAYLLHDVDTGTVGVVDPSEAVPVIDALSRKNRNLTYILNTHHHHDHTGGNVELKARYGAKVIGSGTDKKRIPGIDIHLNDGDKWMFAGHEVRVMDTPGHTQGHISFYFPGSGAIFTGDTLFSLSCGKLFEGTPQQMLSSLKKIMSLPDNTNIYCGHEYTLNNTKFALSIEPENEELQSYAAQVAYLRSKGLPTIPTTLKMEKACNPFLRTSSAAIRQSLNIAATANDAEALGGIRQAKDNF

>GmGLYII-10

LRSQMCSFSTTSFSSSSSKLLFHQLFEKKSSTYTYLLADASHPEKPTLLIDPVDRTVDRDLSLIEQLGLKIVYTMNTHVHADHVTGTGLIKGKVPSVKSVISKASGATVDLYVEPGDKVHIGDLFLEVRATPGHTKGCVTYVTGDAPDQPQPRMAFTGDTLLIRGCGRTGFQIYTCSKLLEQRRKWVKV

>GmGLYII-11

TGKKRIPAIDIHLNDGDKWMCAGHEVRVMDTPGHTQGHISFYFPGSGVIFTGDTFFNLSCGKLFEGTPQQVVLNCTCPFFLFFFF

>GmGLYII-12

MATHRLALIIQNPSNDDEFLLVKQSRPPKFHDEEYDSFVDSDLWDLPSAQLNPLLAESEPPVELELAVSHSESQDVDLRKFDIRSALNEVFGQLGFGAVDGGGWKFHKYVKEAAFGPDLPVNTVFIVGKLVAAEDKDFRDSYRWKSVRSCLNWILEVKPHGDRVGPLVVIGLINESSISTKWKVPPAINYQEYPPGNIIIPMGSRTLRPFHTTNLVVFAPENVSNDSGENNFIVRGDALIVDPGCLSEFYGELEKIVTALPRRLVVFVTHHHPDHVDGLSVIQKCNPDATLLAHEKTMHRISRDVWSLGYTPVTGDEDIDIGGQRLRVIFAPGHTDGHMALLHANTHSLIVGDHCVGQGSATLDIKAGGNMSEYFQTTYKFLELSPHALIPMHGRVNVWPKQMLCGYLKNRRSREANIVKAIEGGAKSLFDIIVYVYSDVDRRAWIAASSNVRLHVDHLAQQHKLPKDFSIQKFKNTCGLHFLSRWIWAYGSGSLSHQIGKSPFLVAGVLAGIAGIAVLYCQRKFTK

>Medtr1g032500.1(GLYII-1.1)

MTIEVLVLGAGQEVGKSCVIVKINGKRIMFDCGMHMRHTDHSRYPDFKKISDSGNFNDALDCIIITHFHLDHVGALAYFTEVCGYSGPVYMTYPTKALSPLMLEDYRKVMVDRRGEEEQFTSDHIAECMKKVIAVDLKQTVQVDEDLQIRAYYAGHVIGAAMFYVKVGDAEMVYTGDYNMTPDRHLGAAQIDRLRLDLLITESTYATTIRDSKYAREREFLKAVHKCVSGGGKVLIPTFALGRAQELRILLDDYWERMNLKVPIYFSSGLTIQANTYHKMLIGWTSQKIKDTYSTHNAFDFKNVHKFERSMLDAPGPCVLFATPGMLIGGFSLEVFKHWAPSEKNLVALPGYCMAGTVGHRLTSGKPTKVDTDPDTQIDVRCQIHQLAFSAHTDSKGIMDLVKFLSPKHVMLVHGDKPKMVSLKERIDSELGIPCSHPANNEIVTISSTQYVNAEASDTFTKNCLNPNFKFQKCSSMDTCNSTLIDRNLTPELQVEDERVADGVLVMENNNNKKAKIVHEDEILLMLDEKKHEV

>Medtr1g050492.1(GLYII-2.1)

MGTSVQVTPLCGVYNENPLSYLVSIDSFNILIDCGWNDHFDPSLLQPLSRVASTIDAVLLSHPDTLHLAALPYAIKHLGLSAPVYSTEPVYRLGLLTMYDHFLSRKQVSDFDLFTLDDIDSAFQTVTRLTYSQNHHLSGKGEGIVIAPHTAGHLLGGTIWKITKDGEDVIYAVDFNHRKERHLNGTVLGSFVRPAVLITDAYNALNNQPYRRQKDKEFGDILKKTLRAGGNVLLPVDTAGRILELILMLESYWADENLNYPIYFLTYVASSTIDYVKSFLEWMSDSIAKSFEQTRENIFLLKNITLLVSKADLDNAPDGPKVVLASMASLEAGFSHDIFVEWGNDVKNLVLFTERGQFGTLARMLQADPPPKAVKVTVSKRVPLVGEELIAYEEEQNRIKKEEALKASLMKEEEFKASQGADNNAIDPMIIDTGNSQPSPEVAVPKNGGYRDVFIDGFVPPSSSVAPMFPCYENITEWDDFGEVINPDDYVIKEEDMDQAANNVGGDLNGKLDESAASLIFDTKPSKVISDERTVQVRCSLVYMDFEGRSDGRSIKNILSHVAPLKLVLVHGSAEATDHLKQHCLKNVCPHVYAPQIEETIDVTSDLCAYKVQLSEKLMSSVLFKKLGEYEVAWVDAEAGKTENDMLSLLPVSGAPHPHKSVLVGDLKLADFKQFLSTKGVPVEFAGGALRCGEYVTVRKVGDATQKGAGSGTQQIIIEGPLCEDYYKIRDYLYSQFYLL

>Medtr1g110300.1(GLYII-3.1)

MATHKLALIIQNPSNQNEFLLIKQSRPPKFNDEEYDSFLDSDLWDLPSVQLNPLQPQSDPPVEVQISVSHSDEFNFSEFDIHSALKEVFGELGFGIVERGEWKFHKYVKEPAFGPGLPVNTVFIAGKLVDDEIKDFSDSYKWMSIQSCLNWLLEVIPHGDRVGPLVVVGLVNDSSVSANWEAPPAINYQEYPTGVILIPMGSRTAKPFHTTNLVVFAPENVPNASKDNQLIVYGDALIVDPGCLSKFHGELKNIVTALPRRLVVFVTHHHRDHVDGLSVIQKCNPDAILLAHENTMRRISRDDWSLGYTSVTGDEDIYIGGQKLKVIFAPGHTDGHMALLHVNTHSLIVGDHCVGQGSALLDINSGGNMSEYFETTYKFLELSPHALIPMHGRVNVWPKQMLCEYLKNRRSREAAILKAIEGGAKTLFEIVAYVYSNVDRRAWIPASSNVRLHVDHLAEQHKLPKEFSIRNFKNTCGLHFLSRWIWGYTSCSIHPRKSSFLIAGVLVGIAVLVHCSAKTKFRK

>Medtr2g006180.1(GLYII-4.1)

MSSVKKRESNGGTINRETEDQLIVTPLGAGNEVGRSCVYMTYKGKTVLFDCGIHPGYSGMAALPYFDEIDPSTVDVLLITHFHLDHAASLPYFLEKTTFKGRVFMTYATKAIYKLLLSDYVKVSKVSVDDMLYDEQDINRSMDKIEVIDFHQTVEVNGIRFWCYTAGHVLGAAMFMVDIAGVRVLYTGDYSREEDRHLRAAETPQFSPDVCIIESTYGVQHHQPRHTREKRFTDVIHSTISQGGRVLIPAYALGRAQELLLILDEYWANHPELQNIPIYYASPLAKKCLTVYETYTLSMNDRIQNAKSNPFAFKHISALSSIDIFKDVGPSVVMASPGGLQSGLSRQLFDMWCSDKKNSCVIPGYVVEGTLAKTILNEPKEVTLMNGLSAPLHMQVHYISFSAHADSAQTSAFLEELNPPNIILVHGAANEMGRLKQKLMTQFADRNTKILTPKNCQSVEMYFNSQKMAKTIGKLAEKTPEVGETVSGLLVKKGFTYQIMAPDDLHVFSQLSTANVTQRITIPYSGAFCVIQSRLKQIYESVEPSVDEESGVPMLLVHDRVTVKHESEKHVSLHWASDPINDMVSDSVVALVLNINRDLPKIVAESDATKIEEENEKKTEKVMQALLNSLFGNVKVGENGKLIINIDGNVAELNKESGEVESENEGLKERVRTAFRRIQSSVKPIPLSAP*

>Medtr2g018660.1(GLYII-5.1)

MATSNGTDDGTPPSESALIFLGTGCSSMVPNVLCLINPSDPPCSVCAQSLSIPPEKNPNYRCNTSMLIDYCGSGSNHNYILIDVGKTFRETVLRWFVHHRIPKIDSIILTHEHADAVLGLDDVRAVQPFSPTNDIDPTPIYLSQHSMDSIEEKFPYLVQKQRKEGQEIRRVAQMAWNIITDDCNQPFFASGLKFTPLPVMHGEDYICLGFLFGEKSRVAYISDVSRIPASTEYVISKSGAGQLDLLILDSLYRTGSHNVHLCFPQTLEIVKRLCPKQTLLIGMTHEFDHHKDNEFLKEWSRREGIPVQLSHDGLRVPINL*

>Medtr2g072190.1(GLYII-6.1)

MLKSQFIKFTPFFPYKPSFSSLSISTTIKLKSQMASYSTSSSSSKLLFRQLFEKESSTYTYLLADASHAEKPAVLIDPVDRTVDRDLSLIQELGLKLVYAMNTHVHADHVTGTGLIKSKVPDVKSVISKASGATADLYVEQGDKIRFGDLFLEVRATPGHTLGCLTYVTGDGPDQPQPRMAFTGDTLLIRGCGRTDFQGGSAEKLYKSIHSQIFTLPKDTLLYPAHDYKGFSVSTVGEEMQYNPRLTKDEETFKNIMANLNLSYPKMIDVAVPANMVCGVQSKTS

>Medtr2g099090.1(GLYII-7.1)

MLSKASTTAMSAFSSCSRVRTGFSVWPNVRQLCFRKGILYGFMRLFSTPYKTLRGGASRSLRVARFCSVANMSSSLQIELVPCLSDNYAYILHDIDTGTVGVVDPSEATPVIDALSKKNRNLNYILNTHHHHDHTGGNVELKARYGAKVIGSATDKERIPGIDIHLNDGDKWMFAGHEVQVMDTPGHTRGHISFYFAGSGAIFTGDTLFSLSCGKLFEGTPQEMQSSLGKIMSLPDDTSIYCGHEYTLNNTDFALKLEPGNKELRSYAGHVASLRSKGLPTIPTTLKMEKACNPFLRTSNAQIRQLLNIPATADDAEALGIIRQAKDNF

>Medtr2g101390.1(GLYII-8.1)

MAQILNFRNFLFLPSYKPTTHFRLRFLSTLVSSSSRRSNINAPPLHLRRRSTTTSTTPMEVEENSSVGFNKRRAEGTENSGLPKKNLQLKVRKLNPINTISYVQVLGTGMDTQDTSPAVMLFFDKQRFIFNAGEGLQRFCTEHGIKLSKIDHIFLSRVCSETAGGLPGLLLTLAGMGDEGMTVNVWGPSDLKYLVDAMRSFIPNAAMVHTKSFGPTFGTESTVKSQSDPIVLVDDEVVKISAIILQPCQIPSQKTDHSIDIADSLNGKKLLAAKPGDMSVVYVCELPEIQGKFDPEKAKALGLRPGPKYRELQLGNSVESDRQKNVMVHPSDVMDPSIPGPVVLVVDCPTESHLEALLSAKSLDTYGDQVGNLPKAGKSVSCVIHLTPESVVCCSNYQNWMKTFSSAQHIMAGHEKKNIEVPILKASARIATRLNYLCPRFFPAPGFWSLPNQNCSKPVSLASSEDSFSAPSNVIYAENLLKFTLRPYVNLGLDRSCIPPKASSSEIIDELLLEIPEVVEAAQHVRQLWEDSSQAKEDSIPLADHSEVIEEPWLSEDGITPACLENIRRDDLEIVLLGTGSSQPSKYRNVTSIYINLFSKGGLLLDCGEGTLGQLKRRYGVSGADDVVRSLSCIWISHIHADHHTGLTRILALRRDLLKGVPHEPVLVVGPRMLKRYLDAYHRLEDLDMLFLDCKHTFEASLADFENDLQETVNSLDLNNNNAEINASKVDSTLFARGSPMQSLWKRPGSPVDKDTVYPLLRKLKGVIQEAGLNTLISFPVVHCSQSYGVVLEAEKRINSVGKVIPGWKIVYSGDTRPCPELIKASRDATVLIHEATFEEGMVLEAIARNHSTTNEAIETGEAANVYRIILTHFSQRYPKIPVINKEHMDITCIAFDLMSINIADLPVLPKVLPYLKLLFRNDMTVDESNDVVVTVDESDDVVDVATSAS

>Medtr3g089020.1(GLYII-9.1)

MATLTSLPPLPHSLLSLRSKPTRLSVSASALSASGNDGSTSRVPQKRRRRIEGPRKSMEDSVQRRMEQFYEGNDGPPLRVLPIGGLGEIGMNCMLVGNHDRYILIDAGIMFPDYDDLGVQKIIPDTTFIRKWSHKIEALVITHGHEDHIGALPWVIPALDSNTPIFASSFTMELIKKRLKEHGIFLPSRLKIFRTKNKFVAGPFEIEPIRVTHSIPDCCGLVLRCSDGTILHTGDWKIDETPLDGKVFDREGLEELSKEGVTLMMSDSTNVLSPGRTTSESVVADSLLRHISASKGRVITTQFASNLHRIGSVKAAADLTGRKLVFVGMSLRTYLEAAWKDGKAPFDPSTLVKAEDIDAYAPKDLLIVTTGSQAEPRAALNLASFGSSHAFKLTKEDIVLYSAKVIPGNESRVMEMMNRISEIGSTIVMGRNENLHTSGHAYRGELEEVLRIVKPQHFLPVHGEYLFLKEHESLGKSTGIRHTAVIKNGEMLGVSHLRNRRVLSNGFISLGKENLQLKYSDGDKAFGTSGELFLDERMRIALDGIIVVSMEIFRPKNLESLAGNTLKGKIRITTRCLWLDKGKLLDALYKAAHAALSSCPVKSPLPHMERTVSEVLRKMVRKYSGKRPEVIAIAIENPGAVFADEINTKLSGKSQVGPGISTFRRSVDEHRKENQSTALQIRDDGIDIEGLLVEIETITTAAEGDLSDSGESDEFWKPFIASSVEKSIKANNGYVSRKEHKSNTKQDDSEDIDEAKSEEMSDSEPESSKSEKKNKWKTEEVKKLIDLRSDLRDRFKVVKGRMALWEEISQSLLADGISRSPGQCKSLWTSLALKYEEIKNGKDSRKNWQYLEDMERILSSDETPATN

>Medtr4g068100.1(GLYII-10.1)

MTDIKERSVLCALPDVRQICFRKGLLYGFSRVFSIPLKTLRGASRSLRVDQFCSVVNISSSLMIELVPCLRDNYAYILYDVDTGTVGVVDPSEAAPVIDALTKKNLNLTYILNTHHHHDHTNGNTELKERYGAKVIGSDVDKERIPGIDIYLSDGDKWMFAGHEVQIMATPGVTQGHISFYFPGSAAIFTGDTLFSLSCGKIYEGTPEQMLSSLKKITSLSDDTSIYCGHEYTLDNSKFALSIDPQNKELQSYASHVAQLRNKGLPTVPTTLKMEKACNPFLRTWSMEIRRKLKVAASADDAEALGVIRQAEDNF*

>Medtr4g103770.1(GLYII-11.1)

MVTCNSISLSHNLHFYTRFHRLHPTRRSHYCRFRSNALPRDTDGAKVVHKRPRRIEGPRKSMEDSVQRKMEQFYEGSDGPPLRVLPIGGLGEIGMNCMLVGNHDRYILVDAGVMFPGDDELGVQKIIPDTTFIKKWSHKIEAVVITHGHEDHIGALPWVIPMLDSQTPVFASSFTMELIRKRLKDHGIFVPSRLKVFRTRKKFVAGPFEIEPITVSHSIPDCCGLVLRCSDGTILHTGDWKIDETPLDGKVFDREALEELSKEGVTLMMSDSTNVLSPGRTMSESVVADALLRHISAAKGRVITTQFSSNIHRLGSLKAAADLTGRKLVFAGMSLRTYLDAAWKDGKVPIDSSTLVKVEDMHAYAPKDLLIVTTGSQAEPRAALNLASYGSSHAFELTKEDTVLYSAKVIPGNESRVMEMLNRISEIGPTIVMGKNECLHTSGHAYRGELEEVLRIVKPQHFLPIHGELLFLKEHELLGKSTGIRHTAVIKNGEMLGVSHLRNRKVLSNGFISLGKENLELKYSDGDKAFGTSSELFIDERLRIALDGIIVVSMEVCRAQSLDSSVENTLKGKIRITTRCLWLDKGKLLDALHKAAHASLSSCPVNCPLAHMEKTVSEMLRKMVRKYSGKRPEVIAVAIENPGAVLATEINTKLSGKSYVGGISTFRNVVHKENQSTKMQMRGMIGMLEFWRRSRMRRTERRHGYIWRTWKAFDNEALAKK

>Medtr5g068440.1(GLYII-12.1)

MKIYHVPCLEDNYSYLIVDESTKEAAAVDPVEPEKVLEASNSLGLTIKFVLTTHHHWDHAGGNEKIKELVPGIKVYGGSIDNVKGCTNALENGDKVHLGADINILALHTPCHTKGHISYYVTGKEDEDPAVFTGDTLFIAGCGKFFEGTAEQMYQSLSVTLGSLPKPTRVYCGHEYSVKNLQFALTVEPDNLRILEKLTWAQNQRQTGQPTIPSTIGDELESNPFMRVDLPAIQEKMGFNSPVEALGELRKVKDNWRG

>Medtr8g017270.1(GLYII-13.1)

MVVTSSIRLLPSSLTSLVHHRSPYSRRLLRPSSISFPLSPIHSLSSNGIGEVDSHVDQSQVIFIGTGTSEGIPRVSCLTNPSTKCPVCTKAAKPGDKNRRLNTSILVRHSNGTGTHNILIDAGKFFYHSALQWFPKFGIRTLDAVIITHSHADAIGGLDDLRDWTNNVQPSIPIYVAKRDFEVMKKTHYYLVDTSVIIPGAAVSALQFNSISEEPFFVHGLKFTPLPVWHGQGYRSLGFRFGNICYISDVSEIPEETYPLLKDCELLIMDALRPDRSSATHFGLPRALEEVRKIQPKRTLFTGMMHLMDHEEVNDYLTKLLESEGLDAQLSYDGLCIAVRL

>Medtr2166s0010.1(GLYII-14.1)

MKNLNWQLGGLRIVALSDGTHPFPVDTVFRDISKDDIRRDLDRAFLEPPVQGSINAFLVDTGTKRILVDSGAGVLYGDCCGKLLANLRAAGYAPEQIDEVLLTHLHKDHVGGIVTNGRMTFPNAVVRVNEIEANYWLDPDNKAQAPAFLASFFDAAAASVAPYIAAGRFRTFRGEATLAPGIRAVPMPGHTPGHTAYLIESGDAGLLAWGDIVHVAAIQLQDPDATVQYDSDADAARRTRRDTLKRVANKRYLVGAAHIAFPGLGHLRRDGEQYDWVPVNYDATPLR
