# Supplementary material for: Genome-Wide Identification of Glyoxalase Genes in Medicago truncatula and Their Expression Profiling in Response to Various Developmental and Environmental Stimuli
Source: Front Plant Sci. 2017 Jun 1;8:836. doi: 10.3389/fpls.2017.00836 (PMC5452422; doi:10.3389/fpls.2017.00836)
Supplement: Supplementary file 3 [file Data_Sheet_3.DOCX]

>OsDJ-1A

MAAQASPPTKKVLVPIVAGTEPVEAAVPIDVLRRAGADVTVASADDGELVVEVMYGVRIVADALVAGGDCAAAHFDLIVLPGGVPGAANLGGCAALEAMVRRHAATGGLYAAICAAPPLALASWGMLNGLKATAHPLFVDKFPPEVAAVDASVVVDASAVTSRGPATSTEFALALVEQLYSKNKAEQIAKEMLVRYDAGYTIDEVNSVQWKCNGTPKVLVPVANGTEEMELITIIDVLRRADADVVVASAENAGVEIVARHGMRIVADTTLDEAAADDQTSSFDLIILPGGTPGAKTMSSNEKLVTLLKKQAAASKPYGAIGAATAHVLEPHGLLEGKKAADQDGGDECESRVVVDGNVITSGGTGTAMEFAVAAVEKLLGRDVAQRVAEGLLFA

>OsDJ-1B

MAMAAASASAMARRAASWPRLLLLSRAFAAAAAEPKRVLVPVADGTEPVEAAATADVLNRAGARVTVATADPAGDDRGLLVEAAFGVKLVADGRVADLEGEAFDLIALPGGMPGSANLRDCKVLEKMVKKQAEQGGLYAAICATPAVTLAHWGLLKGLKATCYPSFMEKFTAEIIPVNSRVVVDRNAVTSQGPATAIEYALALVEQLYGKEKSEEVAGPLYVRPQPGVDYVIDEFNSVEWKCSGTPQVLVPVANGSEEMEALNLIDILRRAGANVTVASVEDKLQVVTRRHKFNLIADIMVEEAAKREFDLIVMPGGLPGAQKLSSTKVLVDLLKKQAESNKPYGAICASPAYVLEPHGLLKGKKATSFPPMAHLLTDQSACDSRVVVDGNLITSKAPGSATEFALAIVEKLFGREKAVSIAKELIFM

>OsDJ-1C

MAPKKVLLLCGDYMEDYEAMVPFQALQAYGVSVDAACPGKKAGDSCRTAVHQGIGHQTYAESRGHNFALNASFDEVNINEYDGLVIPGGRAPEYLAMDEKVLDLVRKFSDAKKPIASVCHGQLILAAAGVVQNRKCTAYPAVKPVLVAAGAKWEEADTMDKCTVDGNLVTAVAYDAHPEFISLFVKALGGSVTGSNKRILFLCGDYMEDYEVMVPFQSLQALGCHVDAVCPDKGAGEKCPTAIHDFEGDQTYSEKPGHDFALTASFDNVDASSYDALVIPGGRAPEYLALNDKVISLVKGFMDKAKPVASICHGQQILSAAGVLQGRKCTAYPAVKLNVVLGGATWLEPNPIDRCFTDGNLVTGAAWPGHPEFISQLMALLGIKVSF

>OsDJ-1D

MLPSSRYLLAPAPLPAMVVRPPPPHPPSRGTSPLARPPLCRAMARAAPSLSAAASTAASSSTTPAKKKVLLPIAMGTEEMEAVILAGVLRRAGADVTLASVEDGLEVEASRGSHIVADKRIAACADQVFDLVALPGGMPGSVRLRDSVILQRITVRQAEEKRLYGAICAAPAVVLMPWGLHKRKKITCHPSFIEDLPTFRTVESNVQVSGELTTSRGPGTAFQFALSFVEQLFGPCKAEDMDNTLLTKVDDNLERSIEVNEIEWSSDHNPHVLIPIANGSEEMEIIMLTDVLRRANVNVVLASVEKSTSIVGSQRMRIVADKCISDASALEYDLIILPGGPAGAERLHKSSVLKKLLKEQKQTGRMYGGICSSPVILQKQGLLQDKTVTAHPSIVNQLTCEVIDRSKVVIDGNLITGMGLGTVIDFSLAIIKKFFGHGRAKGVANGMVFEYPKS

>OsDJ-1E

MATRPLAASTLLPPLRFCSPLKTPPPSPPPPHLRRLQTLTRALASSSSAMASPPAKKVLVPIASGTEPMEAVITVDVLRRAGADVSVASVDPGSAQVGGAWGVKLAADALLDDLADAEFDLISLPGGMPGSSNLRDCKLLENMVKKHAGKGKLYAAICAAPAVALGSWGLLNGLKATCYPSFMDKLPSEVNAVESRVQIDGNCVTSRGPGTAMEYSVVLVEQLYGKEKADEVAGPMVMRPQHGVEFSLKELNSTSWNVGETPQILVPIANGTEEMEATMIIDILRRAKANVVVASLEETLEIVASRKVKMVADVLLDDALKQQYDLILLPGGLGGAQAYAKSDKLIGLIKKQAEANKLYGAICASPAIALEPHGLLKGKKATSFPGMWNKLSDQSECKNRVVVDGNLITSQGPGTSMEFSLAIVEKLFGRERAVELAKTMVFM

>OsDJ-1F

MAPCKKVLMLCGDYMEDYEAAVPFYALAAFGVAVDCVAPGKKPPGDACLTAVHEFLGHDLYTELPGHRFAVTADFAAAAAADASRYDALVVPGGRFVERLSVDPLAVSLVAAFAGEGETATRRRPVVVTCHSQLLLAAAGAMRGVRCTAFFSMRRVVELAGGTWVEPDPLGLCVADGNVLSAIGWPAHGEIIRELLRAMGARVAGGRGQAVLFLCADYVDDYEANVPFRALAGVGCRVEAACPTKRKGEACVTAIYDATPAAASDERRGHNFAVTADWGDVDADRYACVVVPGGRAPELLATRGEAVALVREFAGKGKVVASIDQGHLLLAAVGLLDGRSCASGVATRVVAGLAGAASVRHGGAVADGKLVTAASWPDLAEFIAHIISLLGITVSF

>AtDJ-1B

MASSSLCHRYFNKITVTPFFNTKKLHHYSPRRISLRVNRRSFSISATMSSSTKKVLIPVAHGTEPFEAVVMIDVLRRGGADVTVASVENQVGVDACHGIKMVADTLLSDITDSVFDLIMLPGGLPGGETLKNCKPLEKMVKKQDTDGRLNAAICCAPALAFGTWGLLEGKKATCYPVFMEKLAACATAVESRVEIDGKIVTSRGPGTTMEFSVTLVEQLLGKEKAVEVSGPLVMRPNPGDEYTITELNQVSWSFEGTPQILVPIADGSEEMEAVAIIDVLKRAKANVVVAALGNSLEVVASRKVKLVADVLLDEAEKNSYDLIVLPGGLGGAEAFASSEKLVNMLKKQAESNKPYGAICASPALVFEPHGLLKGKKATAFPAMCSKLTDQSHIEHRVLVDGNLITSRGPGTSLEFALAIVEKFYGREKGLQLSKATLV

>AtDJ-1E

MASAVQKSALLLCGDYMEAYETIVPLYVLQSFGVSVHCVSPNRNAGDRCVMSAHDFLGLELTLNANFDDVTPENYDVIIIPGGRFTELLSADEKCVDLVARFAESKKLIFTSCHSQVMLMAAGILAGGVKCTAFESIKPLIELSGGEWWQQPGIQSMFEITDCVKDGNFMSTVGWPTLGHGIKLLLESLGGKVCSLEKKQASVLFLIGDYVEDYGINVPFRALQALGCKVDAVTPNKKKGEVCATAVYDLEDGRQIPAEKRGHNFFVTASWDDICVDDYDCVVVPGGRSPELLVMNEKAVALVKSFAEKDKVFAAIGQGKLLLAATGVLKGKRCASGKGMKVMVKVAGGEAVMEKGCVTDGKVVTAASATDLPAFLFDLSTALGLTVMF

>AtDJ-1D

MANSRTVLILCGDYMEDYEVMVPFQALQAFGITVHTVCPGKKAGDSCPTAVHDFCGHQTYFESRGHNFTLNATFDEVDLSKYDGLVIPGGRAPEYLALTASVVELVKEFSRSGKPIASICHGQLILAAADTVNGRKCTAYATVGPSLVAAGAKWVEPITPDVCVVDGSLITAATYEGHPEFIQLFVKALGGKITGANKRILFLCGDYMEDYEVKVPFQSLQALGCQVDAVCPEKKAGDRCPTAIHDFEGDQTYSEKPGHTFALTTNFDDLVSSSYDALVIPGGRAPEYLALNEHVLNIVKEFMNSEKPVASICHGQQILAAAGVLKGRKCTAYPAVKLNVVLGGGTWLEPDPIDRCFTDGNLVTGAAWPGHPEFVSQLMALLGIQVSF

>AtDJ-1A

MASFTKTVLIPIAHGTEPLEAVAMITVLRRGGADVTVASVETQVGVDACHGIKMVADTLLSDITDSVFDLIVLPGGLPGGETLKNCKSLENMVKKQDSDGRLNAAICCAPALALGTWGLLEGKKATGYPVFMEKLAATCATAVESRVQIDGRIVTSRGPGTTIEFSITLIEQLFGKEKADEVSSILLLRPNPGEEFTFTELNQTNWSFEDTPQILVPIAEESEEIEAIALVDILRRAKANVVIAAVGNSLEVEGSRKAKLVAEVLLDEVAEKSFDLIVLPGGLNGAQRFASCEKLVNMLRKQAEANKPYGGICASPAYVFEPNGLLKGKKATTHPVVSDKLSDKSHIEHRVVVDGNVITSRAPGTAMEFSLAIVEKFYGREKALQLGKATLV

>AtDJ-1F

MGSMAQKSVLMLCGEFMEAYETIVPLYVLQAFGVSVHCVSPGRKTGDKCVMAAHDLLGLEIYTELVVDHLTLNANFDGVIPDQYDAIIIPGGRFTELLSADEKCVSLVARFAELKKLIFTSCHSQLFLAAAGLLTGGMKCTAFESMKPFIELSGGAWWQQPGVQTLFEITDCVKDGSFMSTMGWPTLGHSLKVLLESLGSKISSSKENHQTSLLFLIGDCVEDYSINVPFKAFQALGCKVDAVTPTKKRGEKCATIVHDLEDGRQLPTEKFGHNFYVTVAWDDVSVDDYDCIVVPGGRSPELLVMNPKAVELVRKFVEKGKFVAAIGMGNWLLAATGALKKKRCASSYGTKVAVKVAGGEIVESERCVTDDKLVTAASTSDLPAFLYALSTALGLSVVF

>AtDJ-1C

MGSLGYSISMIASLSPTLMESRLISSMGCVSMTVAPSFSSVSVVSSSLGTTRRDRTLKLRSSMSPGMVTTLDSDVGVGSSATTKKVLVPIGYGTEEIEAVVLVDVLRRAGADVTVASVEQKLEVEGSSGTRLLADVLISKCADQVYDLVALPGGMPGAVRLRDCEILEKIMKRQAEDKRLYGAISMAPAITLLPWGLLTRKRTTGHPAFFGKLPTFWAVKTNIQISGELTTSRGPGTSFQFALSLAEQLFGETTAKSIEEFLLLRDGYQNPKNKEFNSIDWSLDHTPRVLIPVANGSEAVELVSIADVLRRAKVDVTVSSVERSLRITAFQGTKIITDKLIGEAAESSYDLIILPGGHTGSERLQKSKILKKLLREQHESGRIYGATNSSSTVLHKHGLLKEKRTTVYPSESDEPMNQQMIEGAEVVIDGNVITSLGLATVTKFSLAIVSKLFGHARARSVSEGLVHEYPRQ

>GmDJ-1A.1

MHSINFKRQGGMPGSARLRDCDVLRKITCRQAEENSLYGAICAAPAVSLLPWGLLKKKKVSRGLTTSRGPGTSYQFALSLAEQLFGESVAKEVAELMLMRTDDDNAAKKEFNKVEWSVGHHTPSVLVPIVHGSEEIEVVTVVDILRRAKAKVIVASVEKSLEVLASQGTKIVADILIGDAQESPYDLIILPGGTAGAQRLSKSRILKKLLKEQNSAKRIYGAVYSSLAILQKQGLLKDKRTTAHPSVLVKLKDEEINGAKVDIDGKLITSEVLATVTDFALAIVSKLFGNGRARSVAEGLVFEYPKECM

>GmDJ-1A.2

MGAFEEKEDNLPRFWAIKSNLQVSRGLTTSRGPGTSYQFALSLAEQLFGESVAKEVAELMLMRTDDDNAAKKEFNKVEWSVGHHTPSVLVPIVHGSEEIEVVTVVDILRRAKAKVIVASVEKSLEVLASQGTKIVADILIGDAQESPYDLIILPGGTAGAQRLSKSRILKKLLKEQNSAKRIYGAVYSSLAILQKQGLLKDKRTTAHPSVLVKLKDEEINGAKVDIDGKLITSEVLATVTDFALAIVSKLFGNGRARSVAEGLVFEYPKECM

>GmDJ-1A.3

MQDNLPRFWAIKSNLQVSRGLTTSRGPGTSYQFALSLAEQLFGESVAKEVAELMLMRTDDDNAAKKEFNKVEWSVGHHTPSVLVPIVHGSEEIEVVTVVDILRRAKAKVIVASVEKSLEVLASQGTKIVADILIGDAQESPYDLIILPGGTAGAQRLSKSRILKKLLKEQNSAKRIYGAVYSSLAILQKQGLLKDKRTTAHPSVLVKLKDEEINGAKVDIDGKLITSEVLATVTDFALAIVSKLFGNGRARSVAEGLVFEYPKECM

>GmDJ-1B.1

MSLLLLPQPPTPLSTVTFSAAARAPFAAVTPPRPRTLTPKPALSLSAPITTTAPNNAIPPKKVLVPIGLGTEEMEAVIMIHVLRRAGADVTVASVEPQLQVEAAGGTKLVADTDISACSDQVFDLVALPWQGGMPGSARLRDCDVLRKITCRQAEENRLYGAICAAPAVTLLPWGLLKKKKITCHPAFYDRLPRFWAVKSNLQVSRGLTTSRGPGTTYQFALSLAEQLFGDSVANEVAESMFMRTDDDHAAKEFNKVEWSVGHHTPSVLVPVAHGSEEIEVVTVVDILRRAKAKVIVASVEKSLEVLASQGTKIVADILIGDAQESAHDLIILPGGTAGAQRLSKSRILKKLLKEQNSAERIYGAVCSSLAILQKQGLLKDKRATAHASTLDKLKDKEINGAKVVIDGKLITSEGLATVTDFALAIVSKLFGNGRARSVAEGLVFEYPKK

>GmDJ-1B.2

MSLLLLPQPPTPLSTVTFSAAARAPFAAVTPPRPRTLTPKPALSLSAPITTTAPNNAIPPKKVLVPIGLGTEEMEAVIMIHVLRRAGADVTVASVEPQLQVEAAGGTKLVADTDISACSDQVFDLVALPGGMPGSARLRDCDVLRKITCRQAEENRLYGAICAAPAVTLLPWGLLKKKKITCHPAFYDRLPRFWAVKSNLQVSRGLTTSRGPGTTYQFALSLAEQLFGDSVANEVAESMFMRTDDDHAAKEFNKVEWSVGHHTPSVLVPVAHGSEEIEVVTVVDILRRAKAKVIVASVEKSLEVLASQGTKIVADILIGDAQESAHDLIILPGGTAGAQRLSKSRILKKLLKEQNSAERIYGAVCSSLAILQKQGLLKDKRATAHASTLDKLKDKEINGAKVVIDGKLITSEGLATVTDFALAIVSKLFGNGRARSVAEGLVFEYPKK

>GmDJ-1C.1

MEDYEAMVPFQALQAFGLAIYPRKKSDDVCCTAIHVLADTQTYSETVGHNFALNATFDEVDASSYDGLWVPGGRAPEYLAHIPGVVELVTKFVSLGKQIASICHGQLILAAAGVVEGRKCTLFLLLNQCWLLLAFGGKISGFDKKILFICGDYMEDYEVKDHFQSLQALGSHVDAVCPSKKAGDTCPTAKPGHTFALTATFDDVDPSGYDALVIPGGQAPEYLALNESVIALILSAAGVLKGRKCSAYPAVKLNVVLSGAAWLEPESISRCFTDGNLVTGAAWPGHPEFIAQLMALLGIQVSF

>GmDJ-1D.1

MALRHLRFFPHTLPLTLTPTPNPNNSNRFSFFTPSLSSTTLMATAHKVLVPIADGTEPMEAVITIDVLRRSGADVTVASASDNLAVQALHGVKIIADAPVRDVAATSFDLVALPGGLQGVENLRDCKVLEGLVKKHVEDGRLYAAVCAAPAVVLGPWGLLNGKKATCYPALMEKLAAYAAATSESRVQVDGRVVTSRAPGTTMEFAITLIEQLIGKEKADEVAGPLVMHSNHDDEHTFKEFNPVQWTSDNPPKILVPIANGSEEMEAVIIIDILRRAKAKVVVASVEDKLEIVASRKVKLEADMLLDEAAKLSYDLIVLPGGLGGAQTFANSETLVSLLKKQRESNIYYGAICASPALVLEPHGLLKGKKATAFPVMCNKLSDQSEVENRVVVDGNLITSRGPGTSIEFALAIVEKLFGRKLALELAKAVVFARP

>GmDJ-1E.1

MALRHLRIFPHTLPLTLTPKPKLNNSNRFSFFTSSLSLSSTTLMATAHKVLVPIADGTEPMEAVIIIDVLRRSGADVTVASSSANLAVQALHGVKIIADASVSDVAATAFDLVALPGGLQGDENLRDCKVLEGFVKKHVEDGRLYAAVCAAPAVVLGPWGLLNGKKATCYPALMEKLAAYVAATSESRVQVDGTVVTSRAPGTTMEFAIALIEQLIGKEKAYEVAGPLVMRSNHDDEHTFKEFNSVQWTSDNPPKILVPIANGSEEMEAVIIIDILRRAKAKVVVASVEDKLEIVASRKVKLEADMLLDEATKLSYDLIVLPGGLGGAQTFANSETLVSLLKKQRESNKYYGAICASPALVLEPHGLLKGKKATAFPVMCDKLSDQSEVENRVVVDGNLITSRGPGTSIEFALAIVEKLFGRKLALELANAVVFARP

>GmDJ-1F.1

MAPKKVLLLCGDFMEDYEAMVPFQALQAFGLAVDAVCPGKKSGDVCRTAVHVLAGAQTYSETVGHNFSLNATFDEVDAASYDGLWVPGGRAPEYLAHVPGVVELVTKFVSLGKQIASICHGQLILAAAGVVKGRTCTAFPPVKPVLVAAGAHWVEPDTEAATVVDGDLITAATYEGHPELIRHFVKALGGKISGFDKKILFICGDYMEDYEVKVPFQSLQALGCHVDAVCPSKKAGDTCPTAVHDFEGDQTYSEKPGHTFALTATFDDVDPSGYDALVIPGGRAPEYLALNESVIALVKYFFENKKPVASICHGQQILSAAGVLKGRKCTAYPAVKLNVVLSGATWLEPDPISRCFTDGNLVTGAAWPGHPEFIAQLIALLGIQVSF

>GmDJ-1G.1

MASKRILLLCGDFTEDYEAMVPFQALQAFGLTVDTVCPGRKAGDVCRTAIHGIHGDQTYSEMIGHKFVLNATFDEVDASSYDVLWVPGGRSPEYLSRVPGVLELVTKFVSLGKLIASTCHGPLILAASGVLKGRKCTGFPSLKPVLVDAGADWVDPDTMTTTVEDGGFITSTTYEGQPEIISLLVKALGGKISGTKKKILFICGDFVEDFQAKVPFQSLQSLGCHVDAICPSKFAGDFCPTAVHDFEGDQTYSEKHGHHFDLTVAFDDVDPSDYDALVIPGGRSPEYLSLMDPILDLVRHFFLNNKPVGSIGHGQQILAAAGVLKGRKCTAYPDVKLHVVLSGATWLEPDPISRCFTDGNLVTGAAWQGLPEFIAQLMALLGIRVSF

>Medtr2g078060.1(GLYIII-1)

MALSHIRFFPHTLPSTNFTPKLKLNHNRFFFSPSRSSSSSSSTITAMASNARKVLVPIADGTEPMEAVITIDVLRRSGADVTVASAANRLSVQALHGVKIIADASVSDVVNTAFDLVALPGGVPGVDNLRDSAVLEGLVKKHVEDGKLYAAVCAAPAVVLGPWGLLKGLKATGHPSFMEKLSSYTTSVESRVQLDGRVVTSRAPGTTMEFGVALVEQLLGKEKADEVAGPLVMRSNHADEYTFLELNSVQWTFDNPPKILVPIANGTEEMEAVIIVDILRRAKANVVVASVEDKLEIEASRKVKLQADVLLDEAAKTSYDLIVLPGGIGGAQAFANSETLVNLLKKQRESNKYYGAICASPALALEPHGLLKGKKATGFPAMCSKLSDQSEVENRVVIDGNLITSRGPGTSIEFALVIVEKLFGRKLALEIANATVFASP

>Medtr3g064093.1(GLYIII-2)

MKFCWFQTYIETVGHKFTLNRTFDEIDHTNLVDMLVNSGQEIIACICHGHMIQAAANLLEGPKCTAFPPLKLVLIAAGAFWFEHYVNNCSGW

>Medtr3g064115.1(GLYIII-3)

MELEDMLVNSGQEIIACFCHGHLILAAANLLEGCKCTDFPPLKPVLIAAGAHWVEHLYLALFIALVKPFMENKKPVASICHSQHILAAAGVLKY

>Medtr3g064140.1(GLYIII-4)

MAPKRVLLLCGDFMEDYEGMVPFQALQAFGVSVDAVCPGKKSGDVCRTAVHILSGGQTYTETVGHNFTLNATFDEVDHTSYDGLWLPGGRAPEYLAHIPSVVELVTKFVKSGKEIACICHGHLILAAAGVVEGRKCTAFPPVKPVLVAAGAHWVEPDTMSTTVVDGNLITAPTYEGHPELLRHFLKALGGKISGSDKKILFICGDYMEDYEVKVPFQSLQALGCHVDAVCPSKKAGDTCPTAVHDFEGDQTYSEKPGHNFALTATFDDVDPSGYDALVIPGGRSPEYLSLNEAVIALVKHFMENKKPVASICHGQQILAAAGVLKGRKCTAYPAVKLNVVLSGATWLEPDPISRCFTDGNLVTGAAWPGHPEFIAQLMALLGIQVSF

>Medtr4g085900.1(GLYIII-5.1)

MSFLLLLLPQPSTATRLSPFTISTSTISLKPLSTLSPPRSIPNSTLSISTSPPPTPTNAPPPKKVLLPIGFGTEEMEAVILIHVLRRAGAHVTVASVEPQLQVEAASGTKLVADASISECSDQIFDLIALPGGMPGSARLRDCDALRIITCKQAEENRLFGAINAAPAVTLLPWGLLKRKKITCHPAFFHKLPTFWAVKSNIQVSNGLTTSRGPGTAYMFALTLVEQLFGESIAREVAEFLLMRTDDDNVSKKEFNEIDWSVGHHPPSVLIPIAHGSEEIEVVTLIDILRRAKANVVVASVEKTLGVMASQGTKIVADKLISDIQESAHDLIILPGGTAGAERLSKSRILKKLLKEQNSAGRIYGAVCSSPAILHKQGLLKDKKATAHPSALNKLKDGAVNDAVVVIDGKVITSEGLATVTDFALAIVSKLFGNGRARSVAEGLVFEYPRK*

>E.coli_GLYIII

MTVQTSKNPQVDIAEDNAFFPSEYSLSQYTSPVSDLDGVDYPKPYRGKHKILVIAADERYLPTDNGKLFSTGNHPIETLLPLYHLHAAGFEFEVATISGLMTKFEYWAMPHKDEKVMPFFEQHKSLFRNPKKLADVVASLNADSEYAAIFVPGGHGALIGLPESQDVAAALQWAIKNDRFVISLCHGPAAFLALRHGDNPLNGYSICAFPDAADKQTPEIGYMPGHLTWYFGEELKKMGMNIINDDITGRVHKDRKLLTGDSPFAANALGKLAAQEMLAAYAG

>human DJ-1

MASKRALVILAKGAEEMETVIPVDVMRRAGIKVTVAGLAGKDPVQCSRDVVICPDASLEDAKKEGPYDVVVLPGGNLGAQNLSESAAVKEILKEQENRKGLIAAICAGPTALLAHEIGFGSKVTTHPLAKDKMMNGGHYTYSENRVEKDGLILTSRGPGTSFEFALAIVEALNGKEVAAQVKAPLVLKDLE

> DrosophilaDJ-1

GSHMSKSALVILAPGAEEMEFIIAADVLRRAGIKVTVAGLNGGEAVKCSRDVQILPDTSLAQVASDKFDVVVLPGGLGGSNAMGESSLVGDLLRSQESGGGLIAAICAAPTVLAKHGVASGKSLTSYPSMKPQLVNNYSYVDDKTVVKDGNLITSRGPGTAYEFALKIAEELAGKEKVQEVAKGLLVAYN

>Saccharomyces cerevisiae_Hsp31

MAPKKVLLALTSYNDVFYSDGAKTGVFVVETLHPFNTFRKEGFEVDFVSETGKFGWDEHSLAKDFLNGQDETDFKNKDSDFNKTLAKIKTPKEVNADDYQIFFASAGHGTLFDYPKAKDLQDIASEIYANGGVVAAVCHGPAIFDGLTDKKTGRPLIEGKSITGFTDVGETILGVDSILKAKNLATVEDVAKKYGAKYLAPVGPWDDYSITDGRLVTGVNPASAHSTAVRSIDALKN

>Candida albicans_Hsp31

GSHMVKVLLALTSYNETFYSDGKKTGVFVVEALHPFEVFRKKGYEIQLASETGTFGWDDHSVVPDFLNGEDKEIFDNVNSEFNVALKNLKKASDLDPNDYDIFFGSAGHGTLFDYPNAKDLQKIATTVYDKGGVVSAVCHGPAIFENLNDPKTGEPLIKGKKITGFTDIGEDILGVTDIMKKGNLLTIKQVAEKEGATYIEPEGPWDNFTVTDGRIVTGVNPQSAVKTAEDVIAAFECN

>Caenorhabditis elegans_DJR-1.1

MAQKSALIILAAEGAEEMEVIITGDVLARGEIRVVYAGLDGAEPVKCARGAHIVPDVKLEDVETEKFDIVILPGGQPGSNTLAESLLVRDVLKSQVESGGLIGAICAAPIALLSHGVKAELVTSHPSVKEKLEKGGYKYSEDRVVVSGKIITSRGPGTAFEFALKIVELLEGKDKATSLIAPMLLKL
