# Supplementary material for: Genome-Wide Identification of Glyoxalase Genes in Medicago truncatula and Their Expression Profiling in Response to Various Developmental and Environmental Stimuli
Source: Front Plant Sci. 2017 Jun 1;8:836. doi: 10.3389/fpls.2017.00836 (PMC5452422; doi:10.3389/fpls.2017.00836)
Supplement: Supplementary file 4 [file Data_Sheet_4.DOCX]

**Additional file 4: Details information about the gene expression experiments**

1. Experiment ID: MT-00011

Title: GSE14029: Effects of salt stress on *Medicago truncatula* seedlings

Organism: *Medicago truncatula*

Platform: MT_AFFY_MEDICAGO: Affymetrix Medicago Genome Array.

Number of samples: 18

Experiment design: 3d old Jemalong A17 seedlings were treated with 180 mM NaCl, log_2_ fold change ratio was analyzed at 6 h, 24 h and 48 h of stress as compared to their 0 h expression level.

2. Experiment ID: MT-00013

Title: E-MTAB-2681: Global reprogramming of transcription and metabolism in Medicago truncatula during progressive drought and after rewatering

Organism: *Medicago truncatula*

Platform: MT_AFFY_MEDICAGO: Affymetrix Medicago Genome Array

Number of samples: 54
